# Eurasian spruce bark beetle detects lanierone using a highly expressed specialist odorant receptor, present in several functional sensillum types

**DOI:** 10.1186/s12915-024-02066-x

**Published:** 2024-11-20

**Authors:** Jothi Kumar Yuvaraj, Dineshkumar Kandasamy, Rebecca E. Roberts, Bill S. Hansson, Jonathan Gershenzon, Martin N. Andersson

**Affiliations:** 1 Department of Biology, Lund University, Sölvegatan 37, 223 62 Lund, Sweden; 2 Max Planck Center Next Generation Insect Chemical Ecology, Lund, Sweden; 3 Department of Biochemistry, Max Planck Institute for Chemical Ecology, Jena, Germany; 4 Department of Evolutionary Neuroethology, Max Planck Institute for Chemical Ecology, Jena, Germany; 5 Max Planck Center Next Generation Insect Chemical Ecology, Jena, Germany

**Keywords:** *Ips typographus*, Functional characterization, Olfactory sensory neuron, HEK293 cells, Colocalization, Pheromone

## Abstract

**Background:**

Insects detect odours using odorant receptors (ORs) expressed in olfactory sensory neurons (OSNs) in the antennae. Ecologically important odours are often detected by selective and abundant OSNs; hence, ORs with high antennal expression. However, little is known about the function of highly expressed ORs in beetles, since few ORs have been functionally characterized. Here, we functionally characterized the most highly expressed OR (ItypOR36) in the bark beetle *Ips typographus* L. (Coleoptera, Curculionidae, Scolytinae), a major pest of spruce. We hypothesized that this OR would detect a compound important to beetle fitness, such as a pheromone component. We next investigated the antennal distribution of this OR using single sensillum recordings (SSR) and in situ hybridization, followed by field- and laboratory experiments to evaluate the behavioural effects of the discovered ligand.

**Results:**

We expressed ItypOR36 in HEK293 cells and challenged it with 64 ecologically relevant odours. The OR responded exclusively to the monoterpene-derived ketone lanierone with high sensitivity. Lanierone is used in chemical communication in North American *Ips* species, but it has never been shown to be produced by *I. typographus*, nor has it been studied in relation to this species’ sensory physiology. Single sensillum recordings revealed a novel and abundant lanierone-responsive OSN class with the same specific response as ItypOR36. Strikingly, these OSNs were co-localized in sensilla together with seven different previously described OSN classes. Field experiments revealed that low release rates of lanierone inhibited beetle attraction to traps baited with aggregation pheromone, with strongest effects on males. Female beetles were attracted to lanierone in laboratory walking bioassays.

**Conclusions:**

Our study highlights the importance of the so-called ‘reverse chemical ecology’ approach to identify novel semiochemicals for ecologically important insect species. Our discovery of the co-localization pattern involving the lanierone OSN class suggests organizational differences in the peripheral olfactory sense between insect orders. Our behavioural experiments show that lanierone elicits different responses in the two sexes, which also depend on whether beetles are walking in the laboratory or flying in the field. Unravelling the source of lanierone in the natural environment of *I. typographus* is required to understand these context-dependent behaviours.

**Supplementary Information:**

The online version contains supplementary material available at 10.1186/s12915-024-02066-x.

## Background

Chemical communication among insects using pheromones and other odorants is crucial for their survival and reproduction. Odour molecules are detected primarily by the large family of odorant receptors (ORs) present in olfactory sensory neurons (OSNs) inside the sensilla of the antennae [1]. Together with the highly conserved olfactory co-receptor Orco, the ORs form heteromeric receptor complexes that function as ligand-gated ion channels [2, 3]. To date, many ORs have been functionally characterized primarily from Lepidoptera and Diptera [4–9], whereas only a few have been characterized from the large and diverse order Coleoptera (beetles). To the best of our knowledge, slightly more than 20 beetle ORs have been functionally characterized to date, which restricts our understanding of the functional evolution of the OR family in beetles [10–20]. The majority of the characterized ORs belong to species in the Curculionidae family (*Hylobius abietis* L., *Rhynchophorus ferrugineus* Olivier, *Lissorhoptrus oryzophilus* Kuschel,* Ips typographus* L., and *Dendroctonus ponderosae* Hopkins), which includes the bark beetles and other true weevils, several of which are important pests of trees and other plants [10–13, 15, 19, 20].

Conifer-feeding bark beetles (subfamily Scolytinae) that can infest and kill healthy trees use aggregation pheromones to attract large numbers of conspecifics for mating and allowing them to overcome the host tree’s chemical defences by rapid mass attack [21, 22]. In addition, these bark beetles utilize other chemical cues derived from host plants, non-host plants and microbial symbionts for various ecological purposes [23–26]. The Eurasian spruce bark beetle, *I. typographus*, breeds primarily in Norway spruce (*Picea abies* (L.) H. Karst.) throughout large parts of Europe and Asia. This species, like other bark beetles, normally plays an important role in forest ecosystems by contributing to wood decomposition and nutrient recycling through direct feeding as well as through the action of their associated microbes [24]. However, when beetle populations surpass a critical threshold, *I. typographus* frequently destroys vast areas of healthy forests at enormous ecological and economic costs [21]. Males of *I. typographus* find the host tree and bore into the bark phloem from where they release an aggregation pheromone consisting of (4*S*)-*cis*-verbenol and 2-methyl-3-buten-2-ol [26, 27]. These components act synergistically and attract both sexes to trees. Later in the attack, additional compounds are released, including verbenone and ipsenol, which reduce the attraction to the aggregation pheromone [28]. Pheromone compounds used in sexual communication or aggregation by other sympatric bark beetle species typically also act as anti-attractant cues (i.e. reducing active movement towards the attractive aggregation pheromone, see e.g. [29]) for *I. typographus*, including pheromone compounds produced by *Ips duplicatus* (Sahlberg) (*E-*myrcenol) and *Pityogenes chalcographus* (L.) (chalcogran and methyl-(*E*,*Z*)−2,4-decadienoate) competing for the same host species [30, 31]. When these species co-inhabit the same tree, there is competitive niche displacement, with the larger *I. typographus* colonizing the lower parts of the trunk and the two other smaller species primarily the top branches [32, 33].

Among all bark beetles, *I. typographus* is the most comprehensively studied species in terms of peripheral odour coding, both at the level of OSNs and ORs (e.g. [12, 21] and references therein). A total of 23 classes of OSNs, strongly responding to ecologically relevant compounds, have so far been reported in the scientific literature [24, 25, 34–36]. Nine of the 73 ORs expressed in the antennae have been functionally characterized using in vitro heterologous expression systems, with ORs showing selective tuning towards different bark beetle pheromone compounds, plant odours, or volatiles released by fungal symbionts, respectively [12, 15, 16, 19]. So far, however, the OR with the highest expression in the antennae (ItypOR36; ref [19]) had not yet been targeted for functional characterization. The ORs that are highly expressed in insect antennae are frequently tuned to ecologically important compounds. For example, ORs that detect female-produced sex pheromones are typically highly expressed in the antennae of males, and ORs tuned to host cues are often highly expressed in females, as has been shown in both Lepidoptera and Diptera [4, 37–39]. For species (including bark beetles) that use aggregation pheromones to attract males and females for both mate- and host-finding, it is possible that ORs tuned to such compounds are highly expressed in both sexes. Hence, we expressed ItypOR36 in Human Embryonic Kidney (HEK) 293 cells for functional characterization and hypothesized that this receptor would be tuned to a compound of importance to the ecology of the beetle, such as an aggregation pheromone component.

Using a so-called ‘reverse chemical ecology’ approach, in which investigations start by characterizing functions at the molecular level (i.e. chemosensory gene level) to discover ecologically relevant semiochemicals [40, 41], we reveal that ItypOR36 responds selectively to the monoterpene-derived ketone lanierone (2-hydroxy-4,4,6-trimethyl-2,5-cyclohexadien-l-one). This compound has, to our knowledge, never been reported to be produced by *I. typographus* [25, 42, 43], nor has it been studied in relation to the ecology of this species. In contrast, lanierone is a known aggregation pheromone component, or used as an anti-attractant, in several North American congeners [44–49]. Because high expression of an OR gene at the antennal transcriptome level should translate into a high antennal abundance of OSNs expressing the receptor gene, we performed single sensillum recordings (SSR) and in situ hybridization to further explore the expression of the ItypOR36 gene across the *I. typographus* antennae. In line with the prediction, we identify an abundant OSN class that responds to lanierone, and we show abundant labelling of the ItypOR36 gene across the antennal club surface. Interestingly, the lanierone-specific neurons are co-localized inside the same sensilla as several previously described OSN classes with primary responses to various pheromones, plant- or fungal volatiles in different binary combinations. The presence of lanierone-responsive neurons in several functional sensillum types suggests a novel OSN co-localization principle, different from the less variable OSN grouping principle in, e.g. *Drosophila* [50]. Our behavioural experiments in the field and in the laboratory showed context- and sex-specific responses to lanierone. Whereas further investigations are needed to understand the natural source and ecological role of lanierone for *I. typographus*, this study highlights the importance of ‘reverse chemical ecology’ in identifying behaviourally active semiochemicals and their detection mechanisms*.*

## Results

### The odorant receptor ItypOR36 responds specifically to lanierone

**Fig. 1:**
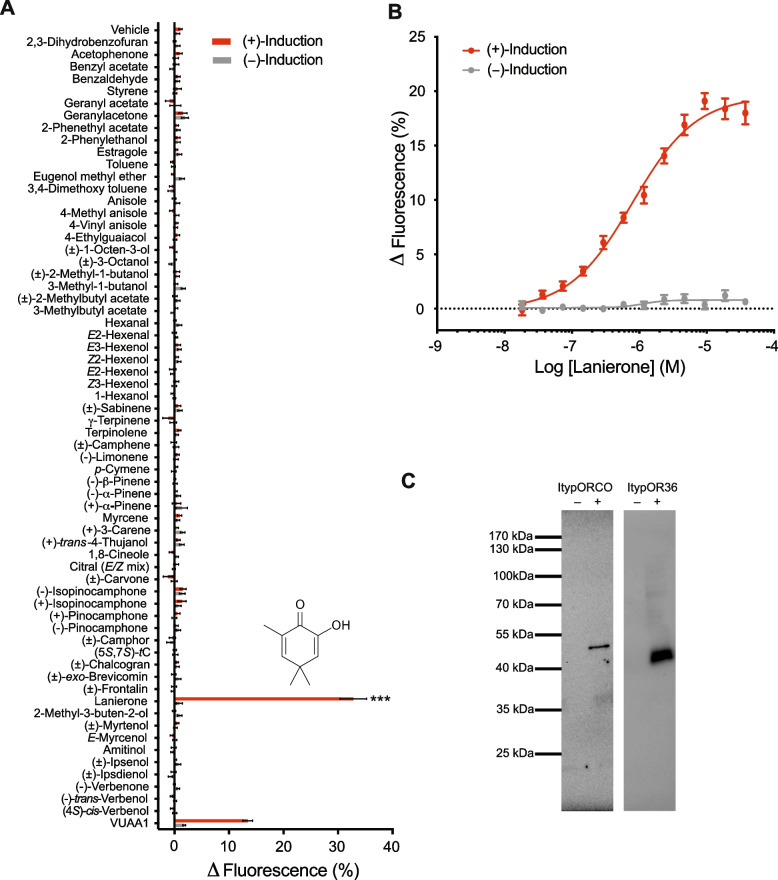
Functional characterization of ItypOR36 in HEK293 cells.** A** Response of HEK293 cells expressing ItypOrco and ItypOR36 to ecologically relevant compounds (30 µM concentration) and the Orco agonist VUAA1 (50 µM). (5*S,*7*S*)-tC = (5*S,*7*S*)-*trans*-conophthorin. Asterisks (***) indicate a significant response to lanierone in induced cells compared to non-induced cells at *p* < 0.001. **B** Dose-dependent response to lanierone. ( +)-Induction: response of cells induced to express ItypOrco and ItypOR36; ( −)-Induction: response of non-induced control cells. Both the screening and the dose response data represent mean responses ± SEM (*n* = 3 biological replicates, each including 3 technical replicates, i.e. *n*_total_ = 9). Raw data from the HEK cell recordings are presented in Additional file 2. **C** Western blots showing the expression of Myc-tagged ItypOrco and V5-tagged ItypOR36 proteins in HEK293 cells. Proteins were only detected from cells induced ( +) to express the Orco and OR36 genes, and not from non-induced ( −) control cells. Uncropped versions of the images are shown in Additional file 1: Fig. S1

The OR with the highest expression in the *I. typographus* antennae, ItypOR36, was selected for functional analysis. Hence, we transfected HEK293 cells with the genes encoding ItypOrco and ItypOR36 and screened this cell line against a panel of 64 ecologically relevant compounds at a concentration of 30 μM. This test odour panel included several pheromones from con- and heterospecific bark beetles, host- and non-host compounds, as well as compounds from microbial symbionts. A large number of these compounds are physiologically and/or behaviourally active in *I. typographus.* In this experiment, ItypOR36 responded only to the monoterpene-derived C_9_-ketone lanierone, with responses only recorded from cells induced to express ItypOrco and ItypOR36, and not from non-induced control cells (not expressing the exogenous receptor genes), indicating proper regulation by the cellular repressor system (F_1,14_ = 209.6, *p* < 0.001; Fig. 1A). The test odour panel included several compounds that are structurally similar to lanierone, such as monoterpenes and oxygenated monoterpenes (both ketones and alcohols), but these compounds did not elicit any response in ItypOR36. Further experiments showed that the response to lanierone was dose-dependent, and the receptor displayed a high sensitivity with an EC_50_ of 790 nM (Fig. 1B). The presence of ItypOrco and ItypOR36 proteins was analysed by Western blot, which showed protein expression of myc-tagged ItypOrco and V5-tagged ItypOR36, as indicated by distinct bands of expected size (Fig. 1C; Additional file 1: Fig. S1). The OR and Orco proteins were detected only in cells induced to express the exogenous Orco and OR genes, and not in the non-induced control cells, again confirming proper regulation by the repression system.

### Abundant olfactory sensory neurons respond specifically to lanierone

**Fig. 2:**
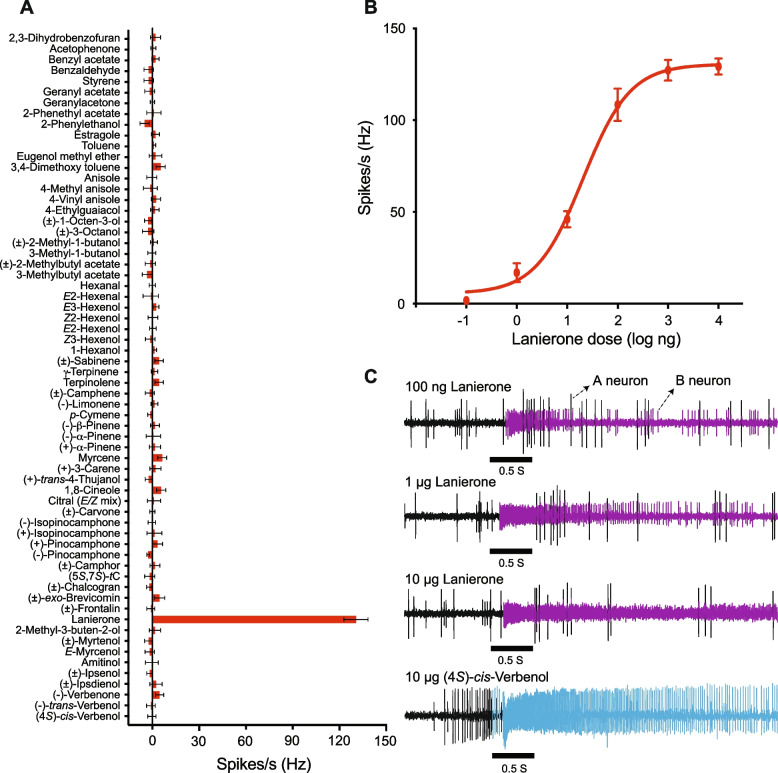
Single sensillum recordings reveal an olfactory sensory neuron (OSN) class responding only to lanierone.** A** Response profile of lanierone-responsive neurons to the test odour panel containing various pheromone compounds, volatiles from host- and non-host plants, as well as fungal symbionts (10 μg dose; *n* = 6). (5*S,*7*S*)-tC = (5*S,*7*S*)-*trans*-conophthorin. **B** Dose-dependent response of the lanierone-responsive OSN class (*n* = 6–9). Responses in A and B are shown as mean ± SEM. **C** Representative action potential traces showing responses to lanierone at 100 ng, 1 μg, and 10 μg doses in the B-neuron (small spike amplitude) and not in the co-localized A-neuron (large spike amplitude), as well as to 10 µg (4*S*)-*cis*-verbenol in the A-neuron (bottom trace). Raw data from the SSR recordings are presented in Additional file 2

**Fig. 3:**
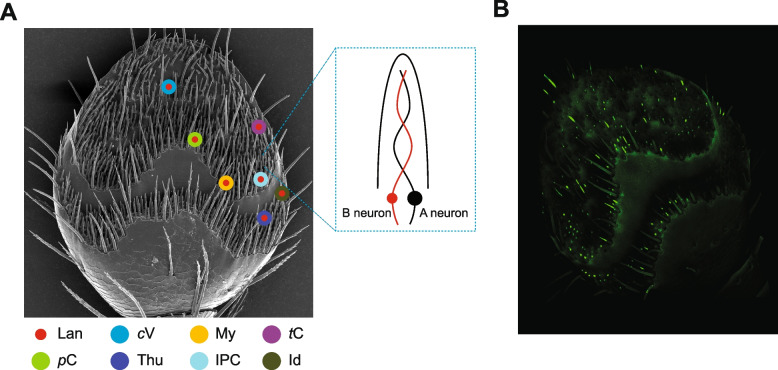
Antennal distribution of the olfactory sensory neuron (OSN) class responding to lanierone and the ItypOR36 gene.** A** Examples of positions of OSNs (B-neurons) responding to lanierone and their co-localization with different A-neurons indicated with differently coloured and sized circles (OSN classed denoted with their primary ligand): red = lanierone (Lan), indigo = (4*S*)-*cis*-verbenol (cV), yellow = myrcene (My), dark green = ( ±)-ipsdienol (Id), blue = ( +)-(1*R*,4*S*)-*trans-*4-thujanol (Thu), light green = *p*-cymene (*p*C), cyan = ( +)-isopinocamphone (IPC), and purple = (5*S*,7*S*)-*trans*-conophthorin (*t*C). For clarity, not all 29 sensilla with a lanierone-responsive neuron are shown. **B** Expression of ItypOR36 across the antenna of *I. typographus*, and shown by Whole Mount-Florescent In Situ Hybridization (WM-FISH) using ItypOR36-specific DIG-labelled antisense RNA probes. Olfactory sensilla expressing ItypOR36 are distributed in all regions of the antennae

Single sensillum recordings from olfactory sensilla of the antennae of *I. typographus* were performed to identify an OSN class tuned to lanierone and to investigate how such neurons are localized across the antenna and their co-localization with other OSN classes inside sensilla. These experiments revealed an OSN class selectively responding to lanierone in both male and female beetles. Sensilla housing the lanierone-responsive neurons were abundant; 29 of the 69 randomly screened sensilla (42%) contained an OSN that responded strongly to lanierone. These sensilla were widely distributed across the antennal club surface. Of all 64 compounds tested, these neurons responded only to lanierone at the 10 µg screening dose (Fig. 2A). Further dose–response assays showed that the lanierone responses were dose-dependent with a response threshold between the 100 pg and 1 ng dose (Fig. 2B). Most olfactory sensilla in *I. typographus* contain two neurons [34]. Our recordings showed that the lanierone responsive neurons are always B-neurons (small spike amplitude), co-localized with (at least) seven previously characterized A-neuron (large spike amplitude) OSN classes (Fig. 2C). Of the 29 sensilla that contained the lanierone-responsive B-neuron, the various co-localized A-neuron OSN classes responded primarily either to *cis*-verbenol (aggregation pheromone component; 10 sensilla), myrcene (1 sensillum), ( ±)-ipsdienol (1 sensillum), (5*S*,7*S*)-*trans*-conophthorin (4 sensilla), ( +)-*trans*−4-thujanol (4 sensilla), *p*-cymene (3 sensilla), or ( +)-isopinocamphone (4 sensilla), respectively. The response specificities of these A-neurons are reported in Additional file 1: Fig. S2-S8 (see also previous studies, ref [24, 34]). Results from in situ experiments targeting ItypOR36 showed that the antennal expression of the ItypOR36 gene corresponds to the locations where the neurons were found in the electrophysiological recordings (Fig. 3A-B), and the abundant labelling is in accordance with both the abundance of the lanierone responsive OSNs and the high(est) antennal expression level of the ItypOR36 gene [19].

### Lanierone is attractive to female *I. typographus* in short-range walking bioassays

**Fig. 4:**
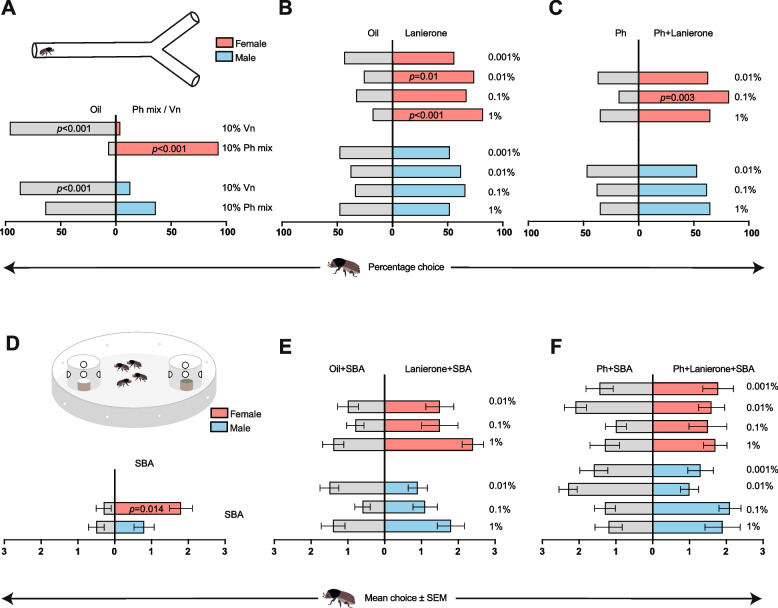
Behavioural effects of lanierone in short-range walking bioassays.** A** Y-tube control experiments showing significant attraction of females to the aggregation pheromone (Ph) mixture (*cis*-verbenol + 2-methyl-3-buten-2-ol, compound ratio 1:50 W/V, dissolved to 10% V/V in paraffin oil), and avoidance of verbenone (Vn, 10%) in both sexes. **B** Y-tube experiments testing four concentrations of lanierone in paraffin oil against blank (paraffin oil control), with significant attraction of females to two concentrations of lanierone. **C** Y-tube experiments testing the Ph mixture (10%) against the Ph mixture plus lanierone, showing significantly enhanced pheromone attraction of females at the intermediate lanierone concentration. **D** Two-choice Petri dish arena control experiment with spruce bark agar (SBA) plugs versus empty traps, showing significant attraction to SBA in females. **E** Two-choice arena experiments with three concentrations of lanierone applied to SBA plugs versus control (SBA plug + paraffin oil). **F** Two-choice arena experiments with the Ph mixture (10%) versus the Ph mixture together with four concentrations of lanierone, both added to SBA plugs. **A–C: ***n*_total_ = 40 beetles; **D–F: ***n* = 10 replicates each including 4 beetles (*n*_total_ = 40 beetles). Raw data from the behavioural experiments are reported in Additional file 2

To investigate short-range (cm-scale) behavioural effects of lanierone on walking beetles, we developed a two-choice Y-tube bioassay. To verify that this newly established bioassay is suitable for testing olfactory-mediated behaviours of *I. typographus*, two control experiments were initially conducted. First, we tested the two-component aggregation pheromone mixture (10% dissolved in paraffin oil; see ‘Methods’ section for further details on pheromone composition) against blank (paraffin oil control). Similar to a previous study using a Petri dish arena assay [25], females were significantly attracted to the aggregation pheromone mixture (*χ*^2^ = 22.53, *p* < 0.001), whereas males did not prefer the pheromone over the blank (Fig. 4A). To verify that the assay is also suitable for testing anti-attractants or repellents, we tested the well-known bark beetle anti-attractant verbenone (10% concentration in paraffin oil) against the paraffin oil blank [29]. Both sexes significantly preferred the blank arm over the verbenone stimulus (males: *χ*^2^ = 12.57, *p* < 0.001; females: *χ*^2^ = 21.16, *p* < 0.001; Fig. 4A).

After verifying the functionality of the bioassay, we next tested different concentrations of lanierone against blank control. Females showed a clear attraction to 1% and 0.01% lanierone (1%: *χ*^2^ = 11.57, *p* < 0.001; 0.01%: *χ*^2^ = 6.26, *p* = 0.01) and a trend for attraction to 0.1% lanierone (*χ*^2^ = 3.0, *p* = 0.083). Males did not show any significant preference across the concentration range of lanierone (Fig. 4B). To investigate whether lanierone has any effect on the response to the aggregation pheromone, the 10% pheromone mix was tested against the same pheromone together with lanierone at different concentrations. Here, females preferred 0.1% lanierone plus the pheromone mix over the pheromone mix alone (*χ*^2^ = 8.9, *p* = 0.003), whereas no significant results were evident for the other lanierone concentrations (Fig. 4C). Males showed no significant preference in this experiment.

We also tested the short-range behavioural effects of lanierone on walking beetles using a still-air trap Petri dish two-choice arena (13 cm diameter) bioassay (described in [25]; see also ‘Methods’ section for additional details). In this assay, the stimuli (dissolved in paraffin oil) were added to spruce bark agar (SBA) plugs that release a semi-natural host odour blend, and the SBA plugs were placed inside small traps inside the arena. First, we tested whether the SBA plugs themselves attract the beetles by placing an SBA plug in one of the traps, leaving the other trap empty. Females, but not males, were significantly attracted to the SBA plugs (*Z* = 2.5, *p* = 0.014, Wilcoxon’s test; Fig. 4D). Next, we applied different concentrations of lanierone (dissolved in paraffin oil) to one of the traps and the equivalent amount of paraffin oil to the other trap (control). Considering the three lanierone concentrations individually, neither males nor females showed any significant preference (Fig. 4E). At each concentration, however, somewhat more females chose the lanierone-baited trap. Due to these consistent trends, we also analysed the female data collectively across the three lanierone concentrations. Altogether, a total of 54 females chose the trap with SBA + lanierone whereas 32 females chose the trap without lanierone. This difference was marginally significant (*Z* = 2.1, *p* = 0.04; related sample Wilcoxon’s test), indicating an overall weak attraction of females to lanierone in this bioassay. When beetles were presented with a choice between aggregation pheromone (10%, added to SBA plug) and the pheromone in combination with different concentrations of lanierone (added to SBA plug), males showed a tendency to prefer the pheromone alone treatment over a mixture containing the pheromone and 0.01% lanierone (*Z* = − 2.2, *p* = 0.028, Wilcoxon’s test; Fig. 4F). However, after Bonferroni correction for multiple statistical comparisons, this result did not pass the threshold for significance. In contrast, no effect of lanierone on pheromone attraction was found in females in this bioassay.

### Lanierone inhibits aggregation pheromone attraction of flying *I. typographus* in the natural environment

**Fig. 5:**
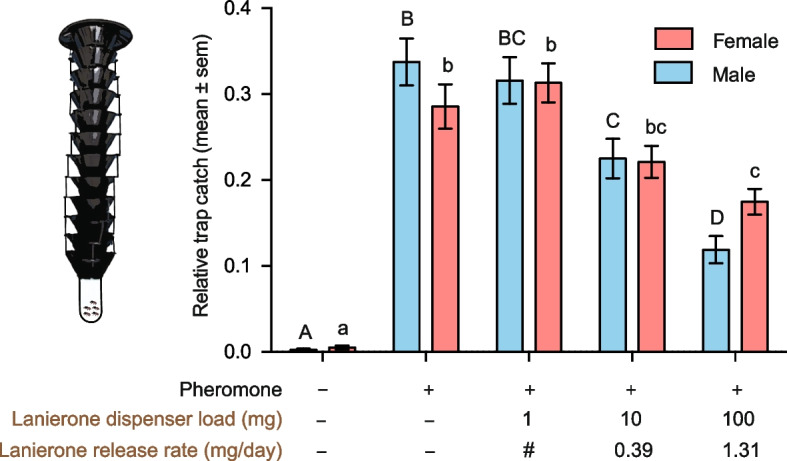
Effects of lanierone on the attraction to the aggregation pheromone in the field. Relative catch of male and female *Ips typographus* in traps baited with aggregation pheromone (Ph) alone, the pheromone combined with three doses of lanierone, and an un-baited control trap in natural field conditions (*n* = 14). Different uppercase (male) or lowercase (female) letters indicate statistically significant differences between treatments at *p* < 0.05. # Note: the release rate from the dispensers loaded with 1 mg lanierone was too low to be detected; hence, both the total dispenser load and daily release rate of lanierone are specified under each bar. Raw data from the field experiment are presented in Additional file 2

Field-trapping experiments were performed to investigate the behavioural effect of lanierone on long-range (meter-scale) flight behaviour in the natural environment of *I. typographus*. In one of the experiments, we tested the effect of three lanierone release rates on the attraction to traps baited with commercial aggregation pheromone dispensers containing *cis*-verbenol and 2-methyl-3-buten-2-ol (see ‘Methods’ for details). A total of 3689 beetles were caught over 14 replicates. The trap baited with only the aggregation pheromone (control) caught a total of 1244 beetles (54% males, 46% females). The addition of lanierone to pheromone-baited traps significantly reduced trap catches of both males and females as compared with catches in traps baited with pheromone alone (GzLM, females: Wald *χ*^2^ = 214.7, d.f. = 4, *p* < 0.001; males: Wald *χ*^2^ = 290.6, d.f. = 4, *p* < 0.001; Fig. 5). The reduction in trap catch depended on the release rate of lanierone, and the effect was stronger on males than females. For males, the intermediate release rate (0.39 mg/day; dispenser load: 10 mg) reduced trap catches by 33% compared to pheromone alone (*p* = 0.043, Hedges* g* effect size = − 1.16), whereas the highest release (1.31 mg/day; dispenser load: 100 mg) reduced trap catches by 65%, suggesting a strong biological effect (*p* < 0.001, Hedges* g* = − 2.55). The trap catches of males were also significantly different between these two release rates of lanierone (*p* = 0.002). For females, only the highest release of lanierone reduced trap catches significantly compared to the pheromone alone treatment, and the biological effect was lower on females than males (39% reduction; *p* = 0.035, Hedges* g* = − 1.37). A non-significant trend for reduced trap catch was observed for females at the intermediate release (23% reduction, *p* = 0.256, Hedges* g* = − 0.75). The lowest dose (dispenser load: 1 mg) of lanierone did not affect the attraction to the aggregation pheromone in any sex.

In another field-trapping experiment where we tested whether lanierone alone is attractive, as few as eight *I. typographus* individuals (all males) were trapped across the four treatments during the 4 days of this experiment: one beetle in the blank control trap, and four, two, and one beetle(s) in the traps with the lowest, intermediate, and highest release of lanierone, respectively. A nearby pheromone monitoring trap caught hundreds of beetles during the same time course, suggesting a high *I. typographus* population density in this area and that the catches in the experimental traps were likely incidental. This interpretation is supported by the observation that the blank traps employed in the experiment with pheromone (described above) also caught one or a few beetles in several replicates.

## Discussion

### Detection of lanierone by a dedicated olfactory pathway

We functionally characterized the most highly expressed OR in the antennal transcriptome of *I. typographus* [19] and hypothesized that it would be tuned to an ecologically important compound, such as a known aggregation pheromone component. Rather than detecting such a compound, ItypOR36 responded to lanierone, which is an aggregation pheromone component produced by some North American *Ips* species [44–49]. The response of HEK cells expressing ItypOR36 was high compared to most previously characterized ItypORs in this system [15, 16, 19], which is in line with the intense band that was detected in our Western blot analysis, suggesting high receptor protein levels in cells [16]. The receptor displayed a remarkable specificity, only responding to lanierone among the 64 tested compounds, most of which are behaviourally and/or physiology active in *I. typographus*, and with several compounds being structurally similar to lanierone. Similar levels of specificity of pheromone responsive ItypORs when expressed in HEK cells have been found for ItypOR46 and ItypOR49, responding only to (*S*)-(-)-ipsenol and (*R*)-(-)-ipsdienol, respectively [19], although the OSNs that putatively express these ORs display secondary responses to a few structurally similar compounds [34]. Also, the sensitivity of ItypOR36 in HEK cells was unusually high. In fact, of all 11 curculionid ORs so far characterized in this system (including ItypOR36), the EC_50_ of ItypOR36 at 790 nM is clearly lower than that for the other ORs, which have EC_50_ values from approx. 2 to 24.5 µM [15, 16, 19]. Whether this is due to the high receptor protein levels in cells providing increased overall sensitivity of the assay, or whether this is an intrinsic property of the receptor itself remains unknown. Three other ItypORs with specific responses to bark beetle pheromones have been functionally characterized previously [12, 19]. These ORs (ItypOR46, ItypOR49, and ItypOR28) belong to an *Ips*-specific OR radiation containing seven ItypORs, and this OR radiation is part of the major coleopteran Group 7 OR subfamily [51, 52], which is particularly expanded in curculionid beetles. Although ItypOR36 is also part of Group 7, its phylogenetic position is rather far from the other known pheromone receptors in *I. typographus* [19, 53]. ItypOR36 is also not closely related to the pheromone receptor RferOR1 in the red palm weevil *R. ferrugineus*, although this receptor falls closer in the curculionid OR phylogeny [10].

Despite previous comprehensive efforts to characterize the response profiles of OSNs in *I. typographus* by SSR [24, 25, 34, 35, 54], lanierone had never been tested and, thus, OSNs responding to this compound had not been identified. The high expression of ItypOR36 made us hypothesize that a putative OSN class detecting lanierone should be highly abundant on the beetle antennae. By random screening of sensilla from males and females specifically searching for lanierone activity, we found that 42% of them contained a small-spiking B-neuron that responded only to lanierone. This relative abundance is more than twice as high as compared to the abundance of the OSN class primarily tuned to the aggregation pheromone component *cis*-verbenol, which previously was suggested to be the most abundant OSN class in *I. typographus* (15% relative abundance [34]). Sensilla containing the lanierone-responsive OSN class were distributed across the antennal club surface, a finding corroborated by our in situ hybridization analysis targeting ItypOR36, and in accordance with the high expression level of the ItypOR36 gene. The antennal distribution of the lanierone OSN class is in contrast to all other described OSN classes in *I. typographus*, which show either a proximal to medial antennal distribution, a medio-lateral distribution, or solely a distal distribution [21, 24, 25, 34]. The high specificity of ItypOR36 in HEK cells was fully recapitulated in the lanierone OSN class, showing no secondary responses even though a high screening dose (10 µg) was used. The matching response profiles between ItypOR36 and the lanierone-specific B-neurons, as well as the correlations between the expression level and antennal distribution of ItypOR36 and the abundance and antennal distribution of the discovered B-neurons, suggest that ItypOR36 is expressed in all of them. However, to conclusively rule out the alternative scenario in which several different lanierone-responsive ORs may be expressed in the seven functional sensillum types, CRISPR/Cas9 or RNAi-based silencing of ItypOR36 should be carried out. Unfortunately, these methods have so far not been developed for bark beetle ORs. However, the fact that no other ItypOR is phylogenetically closely related to ItypOR36 renders such a scenario unlikely, because similar or identical response profiles are most likely to occur among recently duplicated OR paralogs.

Although observed response specificity of ORs and OSNs in part depends on the composition of the test odour panel [55, 56], the lanierone-responsive OSN class is so far the most specific OSN class in *I. typographus*, suggesting that high fidelity detection of this compound is important in this bark beetle. Such highly specific detection of a single compound has, for example, also been shown for the compound geosmin from harmful microbes, activating a dedicated neuronal circuit in *Drosophila melanogaster* (Meigen) [57].

Previous SSR studies have left most of the B-neurons in *I. typographus* uncharacterized as they did not respond to any test compound. Of the 23 previously published OSN classes, only two were classified as B-neurons [34]. The present study shows that many of these previously ‘orphan’ B-neurons belong to the lanierone-responsive OSN class. Based on pioneering studies of Diptera and Lepidoptera, it has traditionally been believed that the same A- and B-neurons (and potential C and D neurons) should always be found together in the same functional types of sensilla [50, 58, 59], although a study of the silkmoth *Bombyx mori* recently reported that a single A-neuron OSN class was paired with two different B-neuron classes [60]. However, our results demonstrate a novel and much broader OSN pairing principle in the bark beetle, with the lanierone-specific OSN class being co-localized in sensilla with seven different A-neuron classes. These A-neurons show primary responses to different bark beetle pheromone compounds (*cis-*verbenol, ipsdienol), plant-derived compounds (*p-*cymene, *trans*-conophthorin, myrcene), or volatiles primarily produced by symbiotic fungi (*trans*−4-thujanol, isopinocamphone), respectively. Previous SSR studies on *I. typographus* and the related ambrosia beetle *Trypodendron lineatum* (Olivier) reported that certain A-neuron OSN classes are sometimes co-localized with a defined responsive B-neuron OSN class, and sometimes with a B-neuron that did not respond to any test compound [61, 62]. The present study is the first to reveal the response in such ‘unresponsive’ B-neurons and to conclusively demonstrate that a single B-neuron class in bark beetles can be present in several functional sensillum types, each housing a different class of A-neuron. The reverse is also true, i.e. that a single A-neuron class can be paired in sensilla with different types of B-neurons. Specifically, it was previously found in *I. typographus* that approximately half of the sensilla housing the A-neuron specific for *cis-*verbenol contain a B-neuron tuned to the host-derived anti-attractant 1,8-cineole, whereas the other half contained an unresponsive B-neuron [61]. In the present study, we show that the previously quiet B-neurons in these sensilla respond to lanierone.

In general, co-localization of OSNs inside insect sensilla has been suggested to improve the ability to assess compound ratios and enable fine-scale spatiotemporal resolution in the detection of ecologically relevant odour mixtures [63–65]. Additionally, strong responses in an OSN may result in peripheral inhibition of the firing activity in the co-localized neuron via passive electrical interactions, which may have behavioural implications [61, 66]. Considering these potential adaptive values of OSN co-localization, it remains unknown whether the several different OSN pairings involving the lanierone-specific neurons in the bark beetle have any functional significance or ecological consequences. Although speculative, it may be possible that selection for increased sensitivity in lanierone detection via increasing the OSN abundance has been favoured at the expense of the potential benefits that a more restricted OSN pairing organization may provide.

Another ‘traditional view’ concerning the organization of the insect olfactory sense has recently been challenged. Specifically, the long-standing belief that insect OSNs (essentially) always express a single chemoreceptor gene along with one or more essential co-receptors has recently been disproven in both *D. melanogaster* and *Aedes aegypti* (L.) mosquitos, with OSNs frequently co-expressing several chemoreceptor genes [67, 68]. Similarly, OSNs that respond to sex pheromone antagonists in the moth *Ostrinia nubilalis* (Hübner) may co-express up to five OR genes, providing the basis for broadly tuned antagonist neurons [69]. Whether beetle OSNs also may co-express multiple chemoreceptors remains to be investigated; however, the high specificity of the lanierone-responsive OSNs that exactly matches the response of ItypOR36 suggests it may be the only ligand-binding chemoreceptor present in these neurons.

### Putative ecological roles of lanierone

What could the ecological role of lanierone be for *I. typographus* considering the observed short-range attraction of walking females in the laboratory and the inhibition of aggregation pheromone attraction among beetles flying in the natural environment? Identifying the (potential) ecological origin of the compound in the natural environment of *I. typographus* is needed to conclusively answer this question and to fully understand these contrasting results. Whereas our field data suggest that lanierone is unlikely to contribute to pheromone-mediated aggregation on trees, the attraction of walking females over short distances could imply that the compound is used in short-range (post-landing) intraspecific sexual communication. If so, we would expect the males to produce the compound, at least under certain circumstances. However, previous chemical analyses of male (and female) *I. typographus* hindgut extracts from different attack phases did not reveal this compound [25, 42, 43]. Because lanierone typically is present in low amounts in the bark beetles that produce it [44], and it may coelute with other more abundant pheromone compounds on certain GC columns [49], it might have been missed in previous studies. Therefore, we extracted pheromones from laboratory-reared male and female *I. typographus* 4 and 10 days after they had been introduced into a spruce log, but we did not detect the compound. However, pheromone production may depend on the physiological status of the insect, as shown for, e.g. *Ips pini* (Say) that requires diapause before they produce any pheromone, including lanierone [44]. Therefore, we performed additional pheromone extractions and volatile collections (solid phase microextraction, SPME) also from individuals that had diapaused in the fridge for 4 weeks (similar to [44]). Male and female beetles were introduced into spruce logs via pre-drilled holes, either individually or a single male together with a single female, and volatiles were sampled from the entrance holes every day for up to 11 days. Again, lanierone was not detected. Collectively, our chemical analyses together with published studies provide no evidence that *I. typographus* produces lanierone. However, whether *I. typographus* does not produce lanierone at all, or only under yet unknown conditions, remains unknown.

Lanierone is a male-produced synergistic aggregation pheromone component in North American *I. pini* and *Ips avulsus* (Eichhoff), and the compound alone is attractive to *Ips integer* (Eichhoff) [47–49]. In *I. pini*, both the lanierone production and its potential to synergize pheromone attraction differ geographically. For example, the compound is produced by beetles from, e.g. New York and Wisconsin where it also has the strongest synergistic effect on pheromone attraction (especially in males), but it is not found in Californian *I. pini* for which the synergistic effect also is the lowest [47, 48]. In addition, different populations of *I. pini* differ in their production of enantiomers of another pheromone component, ipsdienol, and their behavioural responses to pure enantiomers and blends of ( +)- and ( −)-ipsdienol are also population-dependent [70, 71]. The potential existence of *I. typographus* populations that produce lanierone and use it as a pheromone synergist remains to be investigated by analysing populations across its widespread Eurasian distribution. If such populations indeed exist, it may help explaining the attraction of female beetles in our laboratory bioassays, especially if the compound is released when the production of the two-component aggregation pheromone has started to decline [42], that is when the females are walking on the bark.

The inhibitory effect of lanierone on *I. typographus* pheromone attraction in the field was especially strong for the males. Stronger inhibitory effects on males have also been found for other anti-attractant compounds [64, 72], and this is likely because males are the pioneering sex, i.e. the sex that selects a host tree to colonize in the first place. Comparing effect sizes from the field experiment with previous studies of other anti-attractants, the effect of lanierone on males at the release rate of 1.31 mg/day was similar to the effect of other compounds released at much higher rates, e.g., 38 mg/day and 48 mg/day for verbenone and 1,8-cineole, respectively [64]. This suggests that lanierone could be a potent compound to include in anti-attractant blends for improved protection of sensitive trees or forest stands [73]. However, due to the attraction of females in the laboratory, caution is required and the potential use of the compound in forest protection needs further evaluation. Similar inhibition of pheromone attraction by lanierone have been found in the North American *Ips grandicollis* (Eichhoff) and *Ips calligraphus* (Germar), both not producing the compound ([44] but see [46]). As for *I. typographus*, a stronger effect on males than females was shown for *I. grandicollis*, especially when lanierone was combined with ipsdienol, another anti-attractant in this species [44]. Based on the inhibitory effect on pheromone attraction in these two species, it was suggested that lanierone plays a likely role in interspecific pheromone inhibition, reducing competition and the likelihood of interspecific copulations between congeners [44, 46]. Whereas the results from our field experiment and the lack of evidence that *I. typographus* produces lanierone are in line with this idea, the short-range attraction of females is not. To our knowledge, however, short-range behavioural responses to lanierone have not been investigated for *I. grandicollis* or *I. calligraphus*. It also remains unknown whether lanierone is produced by any bark beetle species that is sympatric with *I. typographus*; lanierone was, for instance, not reported from pheromone extracts of the main sympatric competitors *I. duplicatus* or *P. chalcographus* [31, 74–76]*.*

Bark beetle host selection have been proposed to be a sequential process with several different steps, including initial habitat finding followed by host tree selection by flying beetles, and finally host acceptance or rejection after landing [21, 77, 78]. It has been suggested that different semiochemicals are used in the different steps of this process. Our context-dependent behavioural effects of lanierone are in accordance with such a stepwise process, with observed inhibition of pheromone attraction among flying beetles, whereas walking males were indifferent, and females attracted to lanierone at short range. Context- and sex-dependent responses to the aggregation pheromone are also evident, with both sexes being attracted in the field, whereas only females are attracted at short range in the laboratory, as shown in the present and a previous study [25]. This result is expected because both sexes (apart from the first few pioneering males) use the male-produced pheromone to find a suitable host tree; however, after landing the walking females seek out the pheromone-releasing males to get mated, whereas males spread out to avoid competition and attract their own females.

Whichever ecological role lanierone may play for *I. typographus*, it remains puzzling that this compound is detected by the most highly expressed OR in this species. In moths, for example, the major components of female-produced sex pheromones are typically detected by the most highly expressed OR in the male antennae [9, 79–83]. One possible explanation for the high abundance of the lanierone-responsive OSN class may be that lanierone is generally produced in low amounts compared to most other bark beetle pheromone compounds [44, 49], and may thus be present in the environment at very low concentrations. Hence, many OSNs may be required to provide sufficient sensitivity to pick up this signal. In line with this reasoning is the abundance of the two OSN classes that detect the aggregation pheromone components *cis*-verbenol and 2-methyl-3-buten-2-ol, respectively. The male average hindgut quantities of the less volatile *cis*-verbenol and the much more volatile 2-methyl-3-buten-3-ol were shown to be 40 and 500 ng, respectively [42]. Yet, the antennal abundance of the *cis-*verbenol OSN class is much higher than that of the OSN class tuned to 2-methyl-3-buten-2-ol [34], suggesting a negative correlation between pheromone compound concentrations in nature and the abundance of the corresponding OSN class.

Finally, it remains unknown whether the high expression of ItypOR36 could have been affected by the physiological condition of the analysed specimens. The beetles previously used for transcriptomic gene expression analysis [19] and those used for the present SSR were all laboratory reared and had undergone 1 week of storage at 5 °C before they were awakened for experiments. Future studies should investigate whether cold exposure affects OR gene expression and whether individuals in different phases of their life cycle (such as pre- or post-host colonization) express ItypOR36 and other OR genes differentially.

## Conclusions

This study provides novel information of the olfactory detection and behavioural effects of lanierone in *I. typographus*. Lanierone is detected by the most highly expressed ItypOR, which is found in abundant olfactory B-neurons across the antennae. These OSNs are co-localized in sensilla with seven different A-neuron classes; hence, the same A and B neurons are not always paired together in bark beetle olfactory sensilla, suggesting a difference between insect orders in the organization of functional sensillum types. Collectively, our behavioural experiments from the field and the laboratory suggests that the beetles’ response to lanierone differs between sexes and also between flying beetles in the natural environment and walking beetles in the laboratory. Whereas lanierone is produced by allopatric congeners and is likely used in intra-and/or interspecific chemical communication among these species, it remains to be seen whether any bark beetle species that is sympatric with *I. typographus* produces this compound. Also, further investigations are required to understand whether *I. typographus* indeed may produce lanierone but only during specific physiological states or in certain geographical regions. Irrespectively, our study highlights the power of the ‘reverse chemical ecology’ approach in revealing novel insight into the peripheral odour coding strategies in insects and in identifying new bioactive compounds that may have potential for improved pest control and forest management.

## Methods

### Insects

Adult *I. typographus* bark beetles from a continuous culture (originating from Asa, Sweden) reared on natural *P. abies* bolts (length 27 cm, diameter 10–14 cm) under laboratory conditions (described in [84]) were used for single sensillum electrophysiology, laboratory bioassays and in situ hybridizations.

### Cloning and cell line generation

The ItypOR36 gene was amplified from antennal cDNA and cloned into an expression vector as previously described [19, 38]. Briefly, DNA encoding the full-length coding sequence of ItypOR36 was amplified from antennal cDNA (described in [19]) using gene-specific primers containing a V5 epitope tags and 5' and 3' restriction sites (Additional file 1: Table S1). The amplified product was digested using NotI/ApaI and ligated into the expression vector pcDNA5TO (Thermo Fisher Scientific), then transformed into HB101 ampicillin-resistant competent cells and grown overnight in the presence of ampicillin selection antibiotics. Positively transformed colonies were identified by colony PCR as previously described [15], and plasmids containing the ItypOR36 inserts were then purified and Sanger sequenced at the Sequencing Facility, Dept. Biology, Lund University for sequence verification (DNA and amino acid sequences available in Additional file 1). Verified colonies were sub-cultured overnight in LB broth with ampicillin after which large quantities of high-quality pcDNA5TO/ItypOR36 plasmids were produced using a PureLink HiPure Plasmid Midiprep Kit (ThermoFisher) following the manufacturer’s instructions. The ItypOR36 sequence has been deposited in GenBank under the accession number OR166361.

A stable human embryonic kidney (HEK) 293 cell line (originating from ATCC) containing a tetracycline-inducible repressor (TREx) and the ItypOrco gene had been generated in earlier studies, and was cultured in the presence of blasticidin and zeocin antibiotics (New England Biolabs, NEB) as previously described [15, 19, 85]. This cell line was used for further transfection with pcDNA5TO/ItypOR36, with the repressor system allowing us to control (i.e. induce) the expression of the exogenous Orco and OR genes. The plasmid midiprep containing pcDNA5TO/ItypOR36 was linearized using the restriction enzyme FspI (New England Biolabs) and run on a 0.7% TAE agarose gel and purified using a QIAquick Gel Extraction Kit (Qiagen). Transfection reagent Lipofectamine 2000 (15 μL, Life Technologies) and 6 µg of linearized plasmid were each diluted into 500 μL of Optimem medium (Life Technologies) and incubated at room temperature (RT) for 15 min, after which they were mixed and incubated for an additional 60 min at RT. The plasmid/Lipofectamine 2000 mixture was then added to a T-25 cell culture flask containing the HEK293/TREx/ItypOrco cell line at approximately 70% cell confluency and incubated overnight (37 °C, 5% CO_2_). The transfected cells were cultured for approximately 4 weeks in the presence of hygromycin antibiotics (NEB) until a stably expressing TRex/ItypOrco/ItypOR36 hygromycin-resistant cell line had been generated (see [19, 85] for additional details). The newly generated cell line was then passaged three times in the presence of hygromycin (100 μg/mL), zeocin (200 μg/mL) and blasticidin (10 μg/mL) antibiotics, frozen at − 80 °C and thawed prior to functional testing. The cell line expressing ItypOrco/ItypOR36 was cultured for a maximum of 4 weeks or 12 passages without any obvious alterations in growth rate, function, or cell morphology.

### Western blot analysis

The TREx/ItypOrco/ItypOR36 cell line was cultured in the absence of antibiotics for 24 h after which the expression of ItypOrco and ItypOR36 was induced by doxycycline (Sigma), with non-induced cells serving as a negative control for the subsequent Western blot analysis. The day after, the cells were pelleted via centrifugation and proteins extracted as previously described [85]. The following Western blot analysis also proceeded according to previously described methods [4, 81]. Briefly, the proteins were resuspended in 200 µl lysis buffer containing 1X PBS, 2% DDM detergent and 1X protease inhibitor cocktail and incubated at 4 °C for 1.5 h. Samples were then spun at 21,000* g* for 30 min at 4 °C, and the supernatants harvested. Total protein content was quantified using a BioDrop reader according to the manufacturer’s instructions. A total protein amount of 20 µg was mixed with 5X loading solution and incubated at 37 °C for 30 min to denature the proteins before loading onto a 4–15% Criterion™ TGX™ AnykD precast gel (BioRad). The proteins were transferred onto a PVDF membrane using a BioRad Trans Blot Turbo, then blocked with 5% non-fat milk powder in TBST buffer. Primary antibodies (rabbit anti-myc antibody for myc-tagged ItypOrco; rabbit anti-V5 antibody for ItypOR36 (Cell Signalling)) were added at a ratio of 1:2000, and the horseradish peroxidase (HRP)-conjugated secondary anti-rabbit + IgG antibody (Cell Signalling) was added at a ratio of 1:5000 as previously described (8,18). Epitope-containing bands were developed using the Clarity Western ECL-solution (BioRad) and imaged with a BioRad ChemiDoc imaging system.

### Functional characterization of ItypOR36

Functional characterization using HEK293 cells was conducted as previously described [4, 82, 85]. In preparation for the assay, the cells were lifted from the culture flasks and plated (25,000 cells) onto 96-well black-walled poly-D-lysine-coated plates (Thermo Fisher Scientific) and incubated overnight at 37 °C and 5% CO_2_. The following day, the cell culture medium was removed from the plates, and fresh medium was added to the top four rows, and fresh medium containing 1 μg/mL doxycycline induction reagent was added to the bottom four rows to induce the expression of ItypOrco and ItypOR36. The following morning, cells were rinsed with assay buffer (DPBS containing 1 mM probenicid, pH 7.1), then loaded with loading buffer containing the calcium-sensitive fluorophore Fluo4-AM (Thermo Fisher Scientific) and incubated at RT for 30 min. After rinsing and additional 30 min of incubation, cells were tested for response to the odour panel. The odour panel comprised 64 ecologically relevant compounds including bark beetle pheromones from different species, plant odours and fungal volatiles, several of which are primary odorants for previously described OSN classes [24, 25, 34, 35, 54] (Additional file 1: Table S2). The odour compounds were tested at 30 μM concentration using an Omega FluoStar plate reader (BMG Labtech). For the screening assay, test compounds were diluted to a 200X concentration (6 mM) in 100% DMSO (dimethyl sulfoxide), then diluted to 10X (300 μM) in assay buffer. Compounds were diluted to 1X (30 μM) by adding 11 μL of the 10X dilutions to 99 μL of assay buffer in the wells. In addition, a solvent blank (vehicle; 0.5% DMSO in assay buffer) was included as a negative control and VUAA1 (50 µM) that directly agonizes Orco in most insect species [4, 86, 87] was included as a positive control to verify that ItypOrco and the repressor system were functional in each assay.

Background fluorescence was measured in each well immediately before adding a test stimulus. Ligand-induced responses were then measured as the percentage of fluorescence change from background levels 10 s after stimulation. On each plate (biological replicate), readings were taken from three induced and three non-induced wells (technical replicates) per treatment. Cells were tested only once, then discarded. Compounds were regarded as bioactive if they elicited a significantly higher response in the induced cells as compared to the non-induced cells and at least 2% increased fluorescence from the background level. Hence, a general linear mixed model with ‘induction’ as a fixed factor and ‘plate’ (to control for inter-plate variation) as a random factor was performed to determine bioactive compounds. One compound was bioactive and therefore tested further in dose–response experiments. Dose–response curves and EC_50_ values were generated using the non-linear regression function of GraphPad Prism software. Both screening and dose–response experiments were repeated three times (3 biological replicates each including 3 technical replicates; *n*_total_ = 9).

### Single sensillum recordings

Single sensillum recordings were performed following previously described protocols and standard equipment from Syntech (Kirchzarten, Germany) [24, 34, 88]. One antenna from live adult beetles was fixed in dental wax on a coverslip placed on the microscope slide [34]. Tungsten microelectrodes, electrolytically sharpened in KNO_2_, were used for the recordings. The reference electrode was inserted into a pre-made hole in the pronotum and the recording electrode into the base of a sensillum to establish electrical contact with the OSNs. The antenna was exposed to a humidified and charcoal-filtered continuous airstream at 1.8 L/min. To identify a lanierone-responsive OSN class and to estimate its relative abundance and investigate which known OSN classes it may be co-localized with, 69 randomly chosen sensilla across the antennae of 19 beetles (9 males and 10 females) were initially screened with lanierone plus the diagnostic primary ligands of all previously reported OSN classes [24, 25, 34]. After realizing that lanierone-responding OSNs are highly abundant, we screened a subset of them with the same odour panel that was used in the HEK cell recordings with ItypOR36. Additional OSNs were then subjected to dose–response experiments. All compounds were diluted in paraffin oil (w/v) to desired test concentration, and 10 μL of the dilution was applied on filter paper strips (1.5 × 0.5 cm) placed inside Pasteur pipettes capped with 1-mL plastic pipette tips. Odour pipettes were re-filled after every six consecutive stimulations for screening and two stimulations for dose-response assays to avoid bias due to headspace depletion [56]. The compounds were initially tested at the screening dose of 10 μg and dose–response assays were performed at increasing doses from 10 pg to 10 μg. During stimulation, a brief (0.5 s) air pulse at 0.3 L/min (controlled by stimulus controller CS-02, Syntech) was delivered through the stimulus cartridge and into the continuous airstream and onwards to the antenna. Neuronal responses were analysed using AutoSpike v3.3 (Syntech) by counting the number of action potentials (spikes) (separately for the A- and B-neurons, which were distinguished based on spike amplitudes) during the first 0.5 s of the response, and then subtracting the number of spikes during the immediate 0.5 s pre-stimulation period. This net response was then doubled to obtain a value in spikes/s (Hz), and potential blank responses were subtracted.

### Fluorescence in situ hybridization

Fluorescence in situ hybridization was performed following previously described protocols [19, 89]. Briefly, biotin-labelled ItypOR36 was transcribed from linearized recombinant pCS2 + plasmids using the T7 RNA transcription system (Roche). The probe was hydrolyzed to about 800 bp in length with 2 × sodium carbonate buffer (80 mM NaHCO_3_, 120 mM Na_2_CO_3_, pH = 10.2). Freshly dissected beetle antennae were fixed, hybridized and blocked. Biotin-labelled probes were detected by streptavidin horseradish peroxidase conjugates in combination with the substrate FITC-tyramides in a TSA kit (Perkin Elmer, Boston, MA, USA). A control sense probe was included for single probe in situ hybridization. After the washing steps, the antennae were mounted in mowiol mounting media. The slides were visualized on a confocal laser scanning microscope (Leica TCS SP8, Leica Microsystems, Wetzlar, Germany) at the Microscopy Facility, Department of Biology, Lund University.

### Y-tube bioassay

To investigate behavioural effect of lanierone on *I. typographus* in the laboratory, we established a Y-tube olfactometer bioassay. The circular glass Y-tube had the following measurements: main arm length 7.5 cm; choice arm length 5.5 cm; inner diameter 0.48 cm. A rectangular filter paper (1 × 0.5 cm) was inserted inside a clean 1-ml plastic pipette tip, and 20 μL of test odorants dissolved in paraffin oil were added to the filter paper. Immediately, pipette tips containing test compounds were connected to the arms of the Y-tube. The Y-tube received a continuous humidified and charcoal-filtered air supply at 110–130 mL/min via silicone tubes connected to the pipette tips. Male and female beetles were tested separately, one at a time. The Y-tubes were replaced after every ten individuals, and the position of the stimulus/stimuli swapped between arms to control for potential position effects. A total of 40 beetles were used in each experiment. The beetles were considered to have chosen if they crossed two-thirds of the length of one of the choice arms. Beetles that did not choose after 2 min were considered non-responders and discarded from the analysis. The Y-tubes were rinsed with acetone and then baked in an oven at 150 °C between experiments.

To verify whether the bioassay was appropriate to accurately record bark beetle behaviours, two control experiments were performed. First, the two-component aggregation pheromone was tested against blank (paraffin oil control). The two-component aggregation pheromone contained 2-methyl-3-buten-2-ol and (4*S*)-*cis*-verbenol, mixed at a ratio of 50:1 (W/V) and then diluted in paraffin oil to a concentration of 10% (V/V). Secondly, the well-known bark beetle anti-attractant verbenone [29] (10%, dissolved in paraffin oil) was tested against blank in the same manner. We then tested different concentrations of lanierone (0.001–1.0% V/V, dissolved in paraffin oil) alone against blank. Finally, we tested different concentrations of lanierone (0.01–1.0% V/V) added to the aggregation pheromone to investigate whether the presence of lanierone affected the pheromone attraction. Chi-square tests were employed to determine significant preferences for test stimuli, and a Bonferroni correction was employed to maintain the α-level at 0.05, thus, reducing the risk of Type I errors (false positives due to multiple comparisons).

### Trap bioassay

Still-air two-choice trap bioassays were conducted using a previously described Petri dish setup [24, 25]. In this assay, the traps contained spruce bark agar (SBA) plugs, which released a semi-natural host odour blend. First, we tested whether the SBA plugs themselves are attractive to the beetles, by adding an SBA plug to one of the traps and leaving the other trap empty. Next, we tested whether lanierone alone (in the absence of aggregation pheromone) affected the response to the SBA plugs. Lanierone was diluted in paraffin oil to a series of concentrations (0.001–1.0% V/V) and 10 μL was applied to a filter paper disc, placed on top of SBA plugs within treatment traps. A trap containing an SBA plug treated only with paraffin oil was included as a control. Next, to examine the preference of beetles towards the aggregation pheromone mixture compared to a combination of the pheromone and lanierone, we included the same pheromone solution (10% V/V, dissolved in paraffin oil) as used in the Y-tube bioassay. Different concentrations of lanierone (0.001–1.0% V/V) were individually combined with the pheromone. Subsequently, 10 μL of each lanierone-pheromone mixture was added to the SBA plug in the treatment trap, whereas the SBA plug in control trap received 10 μL of the pheromone without lanierone. Males and females were tested separately and placed in the experimental arena immediately after introducing the test odorants. Each bioassay was replicated ten times, with four beetles placed in each arena per replicate. Assays were performed in a dark room with proper ventilation, maintaining a temperature of 23 °C. The beetles’ preference was recorded after 4 h by counting the number of beetles in both control and treatment traps. Beetles that did not enter the traps and remained outside were considered unresponsive and were excluded from subsequent analysis. The choice of beetles in control and treatment traps was statistically analysed using the Wilcoxon’s sign rank test, based on the number of beetles caught in each of the two traps for each of the 10 individual replicates. A Bonferroni correction was employed to maintain the α-level at 0.05, thus, reducing the risk of Type I errors (false positives due to multiple comparisons).

### Field trapping experiments

Field trapping experiments were performed between 21 April and 7 May 2020 in a spruce forest clear-cut in Parismåla, South-East Sweden (N 56° 35′ 15.6″; E 15° 28′ 26.3″). Multiple-funnel traps (WitaTrap, 12 funnel size, Witasek, Austria) were placed with an inter-trap distance of at least 15 m to avoid interference between different treatments, and at least 30 m from the nearest standing spruce tree. Three traps were baited with commercial *I. typographus* aggregation pheromone dispensers (Pheroprax ampulla, Witasek, Austria), each one together with a specific dose of lanierone (1 mg, 10 mg or 100 mg, in commercial bubble cap dispensers; Synergy Semiochemicals, Canada; daily release rates are detailed below). A trap baited with pheromone alone and an un-baited trap served as positive and negative controls, respectively. The baits were hung centrally on the traps, underneath a white plastic cup, protecting the dispensers from direct sunlight and other weather conditions. Treatment positions were initially randomized, and to control for position effects, the traps/treatments were rotated between positions after each replicate according to a temporal Latin square design [90]. A replicate was considered complete when all the five traps collectively had caught > 100 beetles, and the beetles were collected in 15-mL falcon tubes containing 70% ethanol. To investigate whether lanierone alone was attractive to *I. typographus*, a separate experiment with three traps baited with each of the lanierone doses and an un-baited control trap was conducted simultaneously (21–24 April) approximately 350 m away from the experiments that used pheromone dispensers. A pheromone baited trap was placed 40 m from this experiment to monitor *I. typographus* flight activity in this area. In both experiments, captured beetles were counted and sexed based on genitalia dissection in the laboratory.

Dispenser release rates were measured via gravimetry, with aggregation pheromone dispensers releasing [mean ± SD] 66.3 ± 1.9 mg/day (*n* = 3) during outdoor field conditions. Due to the much lower release of lanierone, the dispensers used for release rate measurements were kept in a laboratory fume hood at 24 °C, with the dispensers containing 100 mg lanierone releasing 1.31 ± 0.25 mg/day (*n* = 3), and the dispensers loaded with 10 mg lanierone releasing 0.39 ± 0.08 mg/day (*n* = 3). Release from the 1-mg lanierone dispensers was too low to be accurately measured.

Generalized linear models (GzLM) were used to analyse trap catches [91]. Because the total number of trapped beetles varied between replicates, relative catch (i.e. proportions within replicates) was used for normalization and statistical analysis as in previous studies [23, 64]. Sexes were analysed separately since male and female *I. typographus* often respond differently to attractants and anti-attractants [26, 64, 72]. Goodness-of-fit analysis (Pearson *χ*^2^) and likelihood ratio tests indicated overdispersion in our data (variances larger than the means). We therefore used a negative binomial distribution with log link (IBM SPSS Statistics v23, New York, NY, USA) as the model in our GzLM analyses [92]. Wald *χ*^2^ was used to obtain variance estimates and significance levels, and least significant difference was used in pair-wise comparisons between treatments. ‘Treatment’ (i.e. blank, pheromone control, and pheromone plus each of the three doses of lanierone) was used as a fixed factor in the models, and ‘position’ was included to control for the potential effects of trap position in the field. Furthermore, we used Hedges’ *g* standardized unbiased effect size [93] to investigate the biological effect of lanierone on the attraction to the aggregation pheromone. The effect size is a unitless measure of a treatment effect which is calculated by dividing the difference between the means of a control group (in this case the pheromone control) and a treatment group (the pheromone + lanierone treatments) with the pooled standard deviation for those means.

## Supplementary Information

Additional file 1: Table S1. Sequences of primers used to clone the open reading frame of *I. typographus* OR36 from cDNA and for the incorporation of epitope tags and/or restriction sites for ligation into pcDNA5TO expression vector. Table S2. Compounds used for characterization of *Ips typographus* odorant receptor 36 (ItypOR36) and the lanierone-responsive olfactory sensory neuron class, including their purities, source information, and examples of main biological origins. Fig. S1. Uncropped Western blot images showing protein detection of ItypOrco and ItypOR36 in HEK293 cells, corresponding to the cropped images shown in Fig. 1C. Fig. S2-S8. Response profiles of the seven A-neuron classes and the co-localized lanierone-specific B-neuron class in each of the seven identified functional sensillum types. This file also contains the nucleotide and amino acid sequence of ItypOR36.

Additional file 2. Raw data from HEK cell recordings with ItypOR36, single sensillum recordings, laboratory bioassays, and field trapping experiments.

## Data Availability

All data generated or analysed during this study are included in this published article and its supplementary information files. The data from HEK cell assays, single sensillum recordings, laboratory bioassays, and field experiments are available in Additional file 2. Cloned sequences of ItypOR36 have been deposited in GenBank (accession: OR166361) [94].

## Abbreviations

DMSO: Dimethyl sulfoxide
EC_50_: Effective concentration 50
GzLM: Generalized linear model
HEK cells: Human embryonic kidney cells
Ityp: *Ips typographus*
OR: Odorant receptor
Orco: Odorant receptor co-receptor
OSN: Olfactory sensory neuron
PCR: Polymerase chain reaction
Rfer: *Rhynchophorus ferrugineus*
SBA: Spruce bark agar
SSR: Single sensillum recordings
VUAA1: Vanderbilt university allosteric agonist 1
