# Supplementary material for: Eurasian spruce bark beetle detects lanierone using a highly expressed specialist odorant receptor, present in several functional sensillum types
Source: BMC Biol. 2024 Nov 20;22:266. doi: 10.1186/s12915-024-02066-x (PMC11577813; doi:10.1186/s12915-024-02066-x)
Supplement: Supplementary file 1 — Additional file 1: Table S1. Sequences of primers used to clone the open reading frame of I. typographus OR36 from cDNA and for the incorporation of epitope tags and/or restriction sites for ligation into pcDNA5TO expression vector. Table S2. Compounds used for characterization of Ips typographus odorant receptor 36 (ItypOR36) and the lanierone-responsive olfactory sensory neuron class, including their purities, source information, and examples of main biological origins. Fig. S1. Uncropped Western blot images showing protein detection of ItypOrco and ItypOR36 in HEK293 cells, corresponding to the cropped images shown in Fig. 1C. Fig. S2-S8. Response profiles of the seven A-neuron classes and the co-localized lanierone-specific B-neuron class in each of the seven identified functional sensillum types. This file also contains the nucleotide and amino acid sequence of ItypOR36. [file 12915_2024_2066_MOESM1_ESM.pdf]

Additional file 1

Eurasian spruce bark beetle detects lanierone using a highly expressed specialist odorant receptor, present in several functional sensillum types

Jothi Kumar Yuvaraj, Dineshkumar Kandasamy, Rebecca E. Roberts, Bill S. Hansson, Jonathan Gershenzon and Martin N. Andersson

Material included

Supplementary Table 1	S-2
Supplementary Table 2	S-2 & 3
Supplementary sequences	S-3 & 4
Supplementary Figure S1	S-5
Supplementary Figure S2	S-6
Supplementary Figure S3	S-7
Supplementary Figure S4	S-8
Supplementary Figure S5	S-9
Supplementary Figure S6	S-10
Supplementary Figure S7	S-11
Supplementary Figure S8	S-12

Supplementary Table 1. Sequences of primers used to clone the open reading frame of *I. typographus* OR36 from cDNA and for the incorporation of epitope tags and/or restriction sites for ligation into pcDNA5TO expression vector. All DNA sequences are written in the 5' to 3' direction. Gene-specific coding sequence is in black text; V5 epitope sequences is in blue text; restriction enzyme sites are in red text; Kozak sequence is in bold text. For some primers, four to five additional nucleotides are included on the 5' end of restriction sites to facilitate efficient digestion.

OR	For amplification of full-length coding sequence	For addition of V5 epitope tags and pcDNA5TO restriction sites
ItypOR36	F: ATGGCGAGTGACGAGTTTATAAAAATCCC	F: AGAATGCGGCCGC**CACC**ATGGGCAAGCCTATCCCTAATCCTCTGCTGGGCCTGGACAGCACCGCGAGTGACGAGTTTATA
		R: TTGCGGGCCCTTAGTTGTAGTTATTCATC

Supplementary Table 2. Compounds used for characterization of *Ips typographus* odorant receptor 36 (ItypOR36) and the lanierone-responsive olfactory sensory neuron class, including purities, source information, and examples of main biological origins.

Compound	Purity (%)*	Source*	Examples of biological origin
Acetophenone	99	Acros	Beetle, fungi
Amitinol	91	R. U.	Beetle
Anisole	>99	Sigma-Aldrich	Fungi
Benzaldehyde	>99	Kebo	Non-host, fungi
Benzyl acetate	>99	Aldrich	Fungi
(±)-*exo*-Brevicomin	99	W. F.	Beetle, fungi
(±)-Camphene	95	Aldrich	Host
(±)-Camphor	97	Aldrich	Host, fungi
(+)-3-Carene	99	Aldrich	Host
(±)-Carvone	>99	Fluka	Fungi
(±)-Chalcogran	90	Celamerck, GmbH	Beetle
(±)-1,8-Cineole	99	Aldrich	Host
Citral (*E*/*Z* mix)	99	Aldrich	Fungi
(5*S*,7*S*)-*trans*-Conophthorin	94	W. F.	Non-host, fungi
p-Cymene	>99	Acros	Host
2,3-Dihydrobenzofuran	99	Aldrich	Fungi
3,4-Dimethoxytoluene	98	Givaudan-Roure	Host, fungi
Estragole	>99	Aldrich	Host, fungi
4-Ethylguaiacol	98	Sigma-Aldrich	Fungi
Eugenol methyl ether	>99	Fluka	Host, fungi
(±)-Frontalin	>99	Synergy Semiochemicals	Beetle
Geranyl acetate	97	Aldrich	Fungi
Geranylacetone	>99	Fluka	Non-host, fungi
Hexanal	96	Sigma	Non-host
1-hexanol	>99	Fluka	Non-host, fungi
*E*2-hexenal	98	Aldrich	Non-host
*E*2-hexenol	96	Aldrich	Non-host
*E*3-hexenol	98	Aldrich	Non-host
*Z*2-hexenol	95	Aldrich	Non-host
*Z*3-hexenol	98	Aldrich	Non-host
(±)-Ipsdienol	94	Bedoukian	Beetle
(±)-Ipsenol	95	Synergy Semiochemicals	Beetle
(+)-Isopinocamphone	>99	R. U.	Host, fungi

(-)-Isopinocamphone	>99	R. U.	Host, fungi
Lanierone	>99	Synergy Semiochemicals	Beetle
(-)-Limonene	>99	Fluka	Host
4-Methyl anisole	>99	Fluka	Fungi
(±)-2-Methyl-1-butanol	>99	Aldrich	Fungi
3-Methyl-1-butanol	99	Aldrich	Fungi
(±)-2-Methyl-3-buten-2-ol	>99	Acros	Beetle, fungi
(±)-2-Methylbutyl acetate	>99	SAFC	Fungi
3-Methylbutyl acetate	97	Sigma-Aldrich	Fungi
Myrcene	95	Sigma-Aldrich	Host
E-Myrcenol	>99	Fytofarm	Beetle
(±)-Myrtenol	96	G. B.	Beetle, fungi
(±)-3-Octanol	97	Sigma-Aldrich	Non-host, fungi
(±)-1-Octen-3-ol	98	Janssen Chimica	Non-host, fungi
2-Phenethyl acetate	>99	Aldrich	Fungi
2-Phenylethanol	>99	Sigma	Beetle, fungi
(+)-α-Pinene	98	Janssen Chimica	Host
(-)-α-Pinene	>99	Fluka	Host
(-)-β-Pinene	92	Fluka	Host
(+)-Pinocamphone	84	R. U	Host, fungi
(-)-Pinocamphone	81	R. U.	Host, fungi
(±)-Sabinene	97	Chemos GmbH	Host, non-host
Styrene	>99	Fluka	Fungi
γ-Terpinene	97	Aldrich	Host
Terpinolene	98	Fluka	Host
(+)-*trans*-4-Thujanol	97	Sigma-Aldrich	Host, fungi
Toluene	>99	Merck	Fungi
(-)-*cis*-Verbenol	99	Borregaard	Beetle
(-)-*trans*-Verbenol	97	SciTech Ltd., Prague	Beetle
(-)-Verbenone	>99	Fluka	Beetle, fungi
4-Vinyl anisole	97	Aldrich	Fungi
VUAA1	98	Sigma-Aldrich	None

* Abbrevations: R. U. = gift from Rikard Unelius (Linnaeus University, Kalmar, Sweden); W. F. = gift from Wittko Francke (University of Hamburg, Germany); G. B. = gift from Gunnar Bergström (University of Gothenburg, Sweden).

>ItypOR36_nt sequence

ATGGCGAGTGACGAGTTTATAAAAATCCCTAAAATTTTCCTGATTTTAGGGGGTT
TTTGGCCCTTTTCAGTTACCAAAGACCCGCTTAAAGGTAAACTGTATAAAGTATA
CAGCAAATTCCAGACTTATTCCTACGTGACTTTGATTGCATCTTTGGTCCTAAAAT
TGGTAATTTTGATAGTGCGTAAAGAGGATGAGACGCTAATTTTTCGCGACCTAAA
CGTGTTGGTACTGGTTTTTGAGACCTGCGTTAAAATCCTAATTTTCCAAAGGATGA
AAATCCCACACATGTTCGAACACGCAATGAAACACGAACGGAACATATTCAAAT
CGCGTGATCTAGAGCTGATTATCTGTTACCAAGAGCAGGTCAAATACGGCCGTAG
GGTGAATCTGAGCCAGTTTTTGGTTACGACCCTATCAACGTCCACGTTTGCTGTCA
CCGCCCTTTTGGACGTTTATTGGGCTGCGGACATGTCAAAGTACGAGAAAGAGCC
CTTTATGCACGATCTGTGGTTTCCGTTTCGGCGTGAGACGCACATGAACTGGGTA
ATTTTTTTCAATCTATTCATGATCGTTCAGGGTACCTGCTTCAACACTGCCACTCA
GGCCACTTTGATCAATCTAATGATTTACTCCAGCTCTAGATTGAAGCTTTTAGGGC
TGAAATTAAGAAAGTTTGATGCTATAGCTAGTCAAAATGGTCGGGAAATCCTGGA
GACTGTTCATGATTTAATATTTGAACATCAGGACTTACTTAGTTTTGTAGAGTCTT
TAAATGTTCGCATTAAATACGTTTTATTGATGGAATTTATTTTGAACGAACTCGGT

TTAGCTTCTGGTATTATACAGCTCATAGTGATTGACACGACTTCTTACATGGTATC
AGTAGTAACCATAATTATTTTGCAACTTTTTCAAATATTTGTTATCGCCTGGACTG
CCAACGAAATTACTATTCAGGGAGCTAAGATTGCCGACTCAGTAATGGCAAGCA
ACTGGGTAGAACAGCCAACCAATATCAAAAAATTGTTCCTTATAATGGTGATGAG
AGCCCAGCGCCCTTTAGGGCTAACAGCCGGACCTTTTTTCAATATGAACGCAAAT
ACCGCTGTTTCGACCGTCAAGGCAGCTTACACCTATTTAACCTTTATGATGAATAA
CTACAACTAA

>ItypOR36_AA sequence

MASDEFIKIPKIFLILGGFWPFSVTKDPLKGKLYKVYSKFQTYSYVTLIASLVLKLVILI
VRKEDETLIFRDLNVLVLVFETCVKILIFQRMKIPHMFEHAMKHERNIFKSRDLELIIC
YQEQVKYGRRVNLSQFLVTTLSTSTFAVTALLDVYWAADMSKYEKEPFMHDLWFPF
RRETHMNWVIFFNLFMIVQGTCFNTATQATLINLMIYSSSRLKLLGLKLRKFDAIASQ
NGREILETVHDLIFEHQDLLSFVESLNVRIKYVLLMEFILNELGLASGIIQLIVIDTTSYM
VSVVTIIILQLFQIFVIAWTANEITIQGAKIADSVMASNWVEQPTNIKKLFLIMVMRAQ
RPLGLTAGPFFNMNANTAVSTVKAAYTYLTFMMNNYN

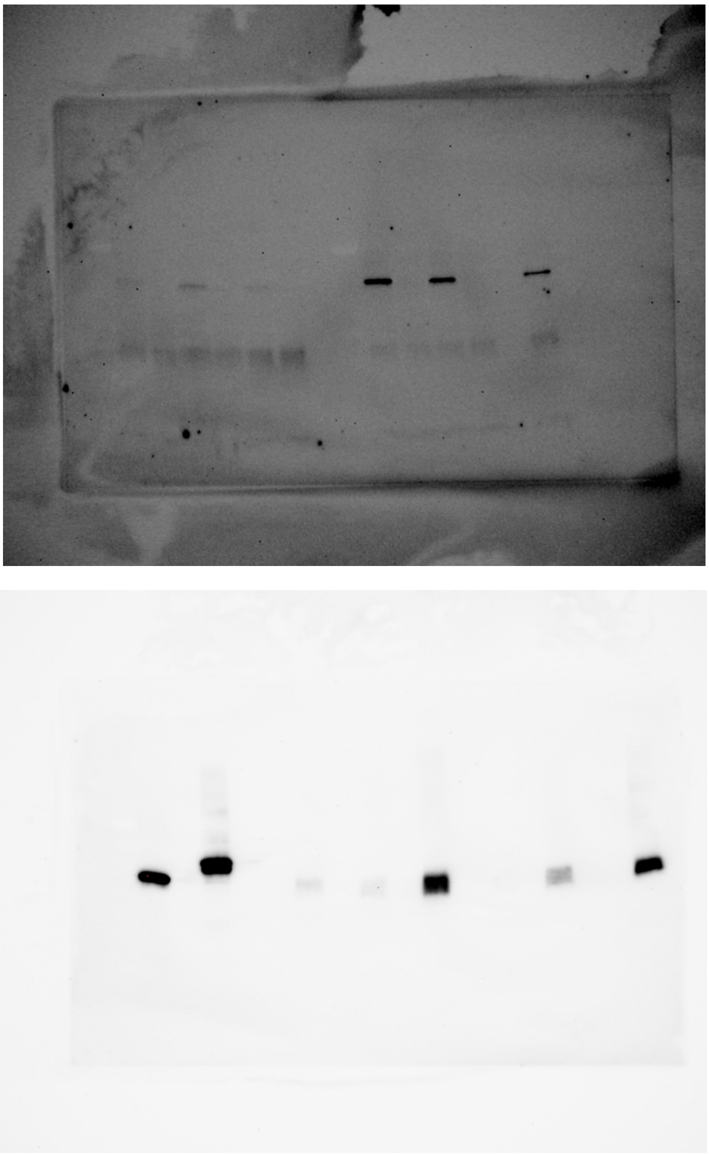

Supplementary Figure S1. Uncropped Western blot images showing expression of Myc-tagged ItypOrco (upper blot) and V5-tagged ItypOR36 proteins (lower blot) in HEK293 cells that were induced to express the exogenous receptor genes. The bands to the furthest right in both blots show Orco and OR detection from the ItypOrco/ItypOR36 expressing cell line, and these bands are shown as cropped images in Figure 1C. The other bands show protein detection from other ItypOrco/ItypOR cell lines, irrelevant for the present study.

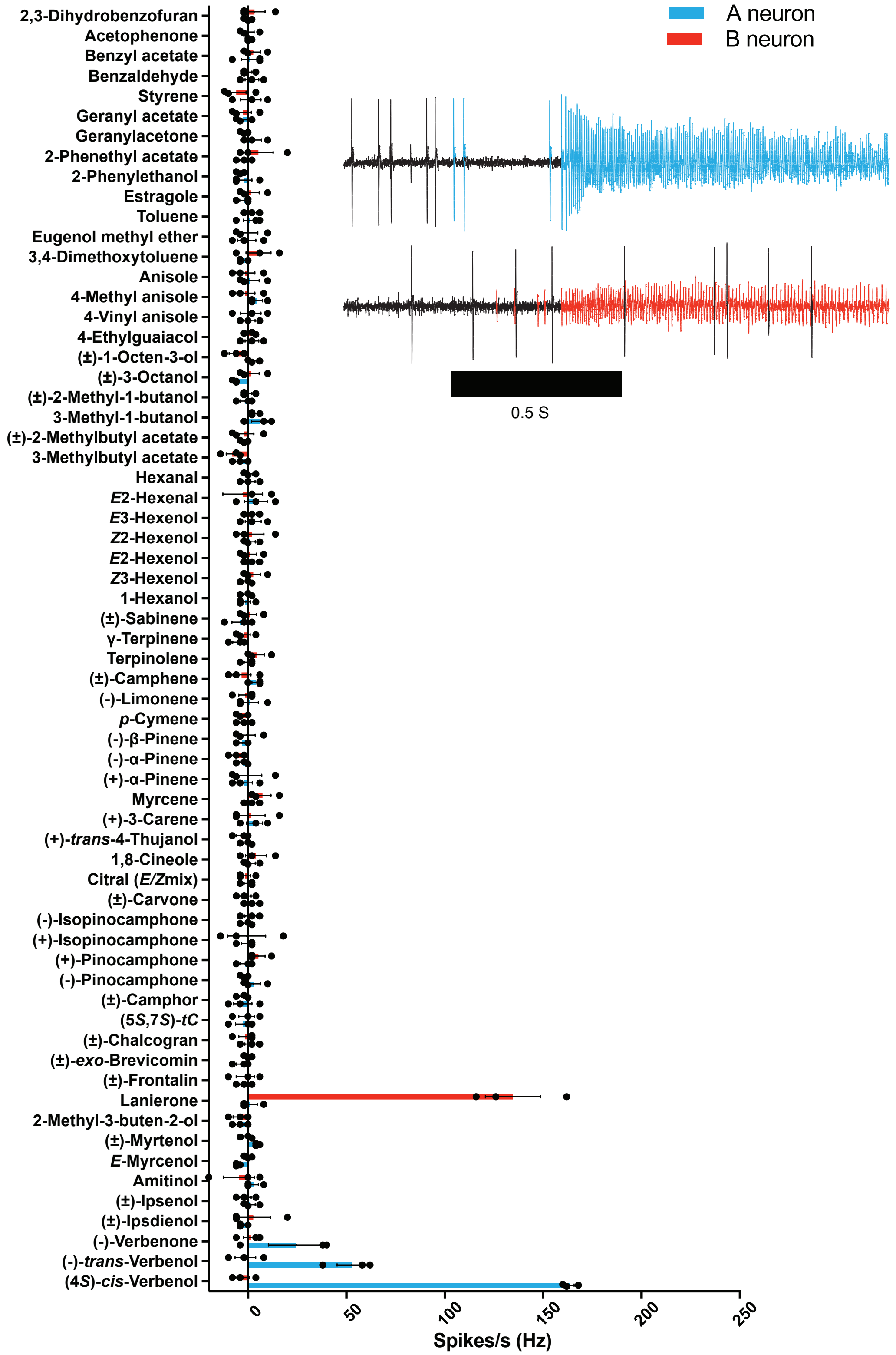

Supplementary Figure S2. Response spectra of the (4*S*)-*cis*-verbenol A-neuron OSN class (blue bars; N=3) and the co-localized lanierone-responsive B-neuron (red bars; N=3) at the 10 µg dose. Error bars show SEM; black dots represent individual data points. Representative action potential traces with response to (4*S*)-*cis*-verbenol in the A-neuron (large spike amplitude; blue spikes) and lanierone in the B-neuron (small spike amplitude; red spikes) are also shown. Raw data are reported in Additional file 2.

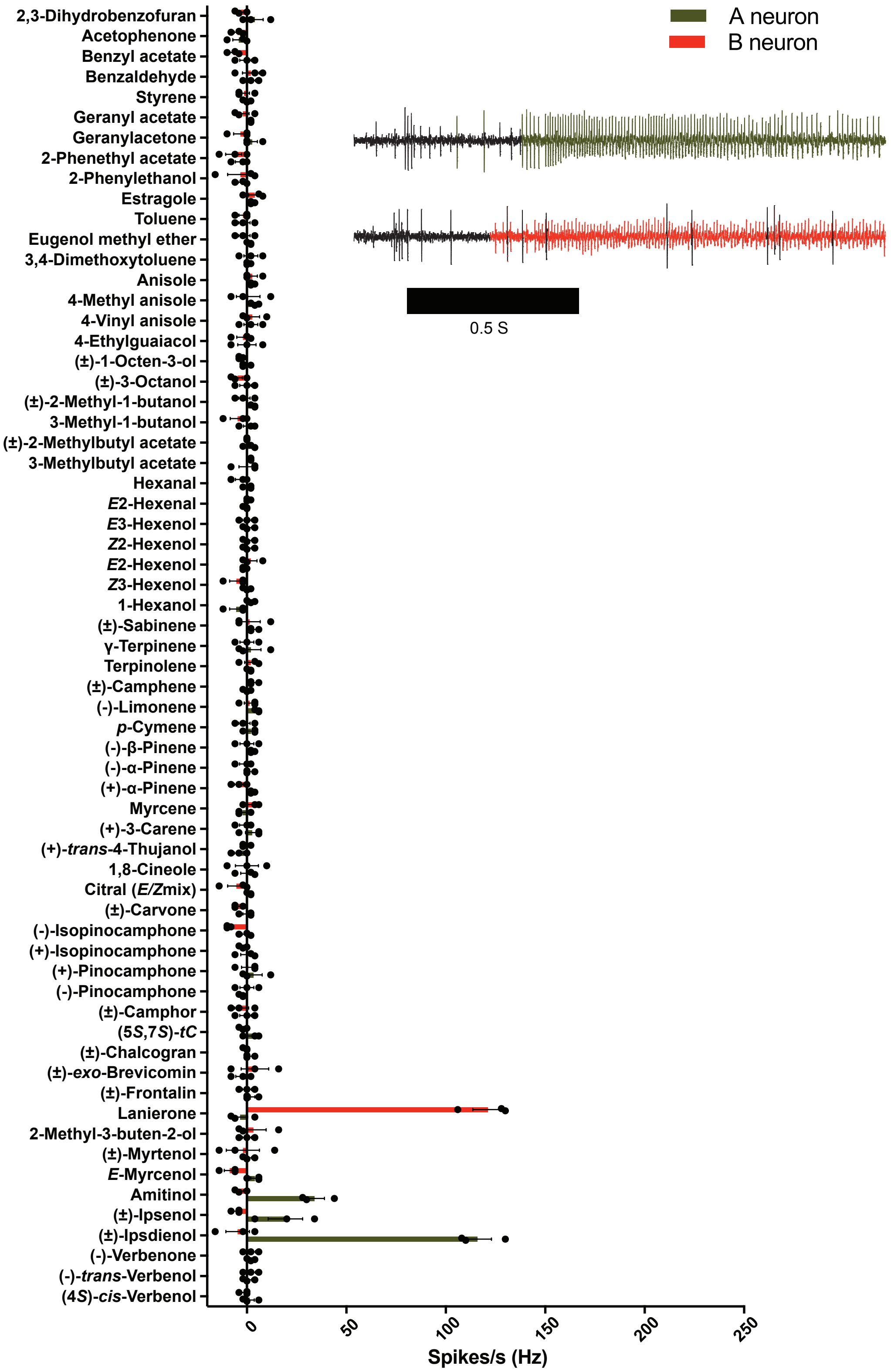

Supplementary Figure S3. Response spectra of the ipsdienol A-neuron OSN class (dark green bars; N=3) and the co-localized lanierone-responsive B-neuron (red bars; N=3) at the 10 µg dose. Error bars show SEM; black dots represent individual data points. Representative action potential traces with response to ipsdienol in the A-neuron (large spike amplitude; dark green spikes) and lanierone in the B-neuron (small spike amplitude; red spikes) are also shown. Raw data are reported in Additional file 2.

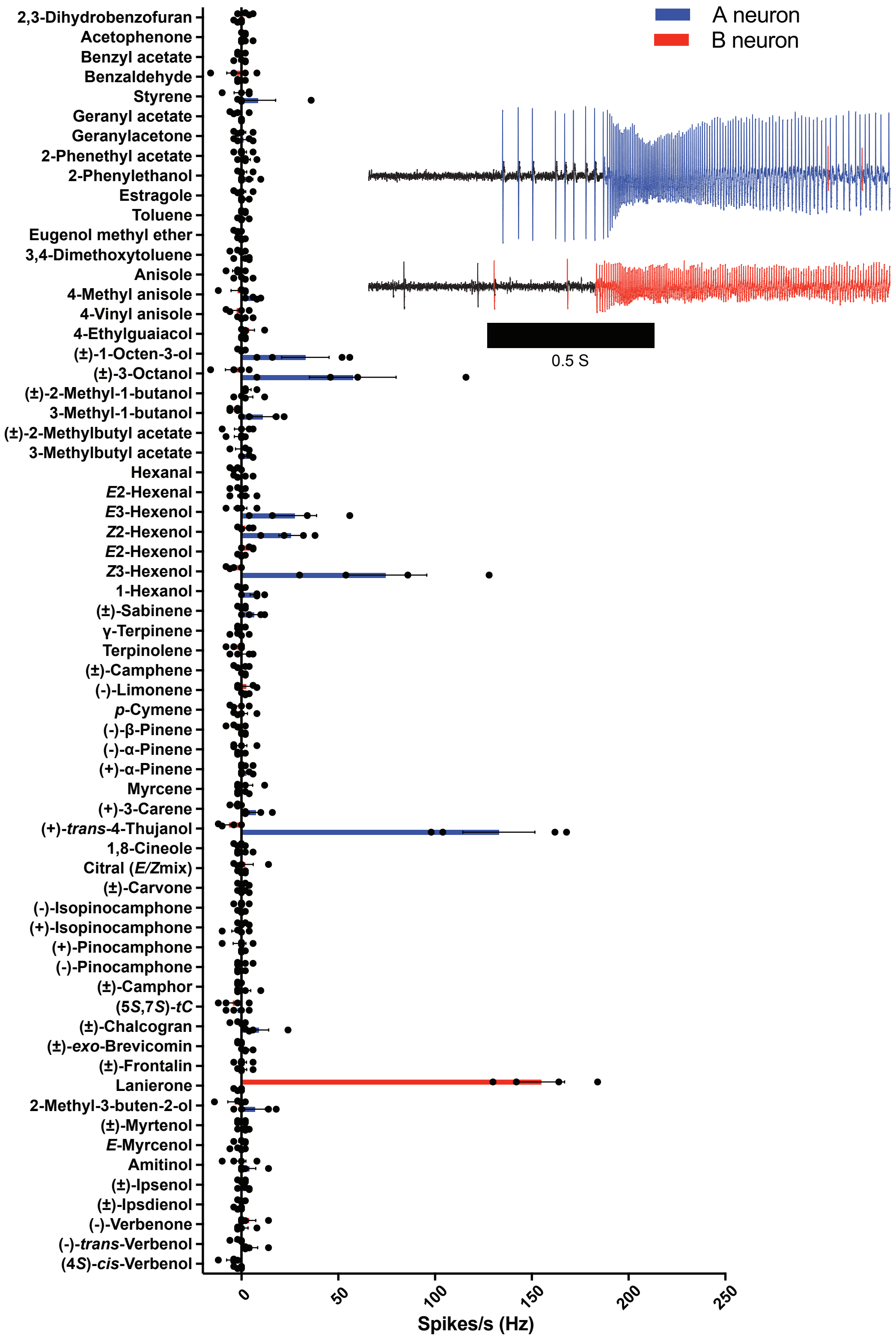

Supplementary Figure S4. Response spectra of the (+)-*trans*-4-thujanol A-neuron OSN class (dark blue bars; N=4) and the co-localized lanierone-responsive B-neuron (red bars; N=4) at the 10 µg dose. Error bars show SEM; black dots represent individual data points. Representative action potential traces with response to (+)-*trans*-4-thujanol in the A-neuron (large spike amplitude; dark blue spikes) and lanierone in the B-neuron (small spike amplitude; red spikes) are also shown. Raw data are reported in Additional file 2.

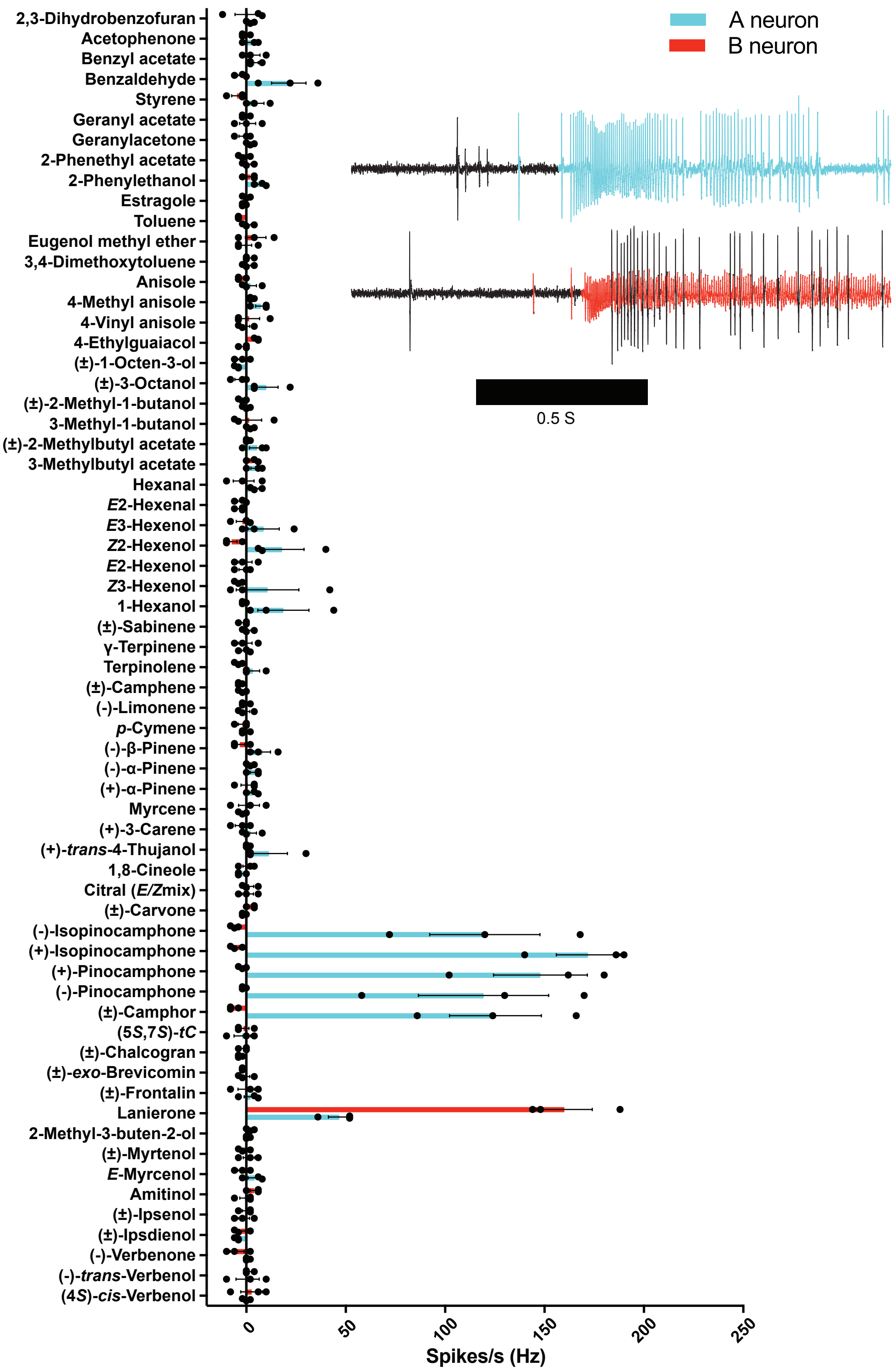

Supplementary Figure S5. Response spectra of the (+)-isopinocamphone A-neuron OSN class (cyan bars; N=3) and the co-localized lanierone-responsive B-neuron (red bars; N=3) at the 10 µg dose. Error bars show SEM; black dots represent individual data points. Representative action potential traces with response to (+)-isopinocamphone in the A-neuron (large spike amplitude; cyan spikes) and lanierone in the B-neuron (small spike amplitude; red spikes) are also shown. Raw data are reported in Additional file 2.

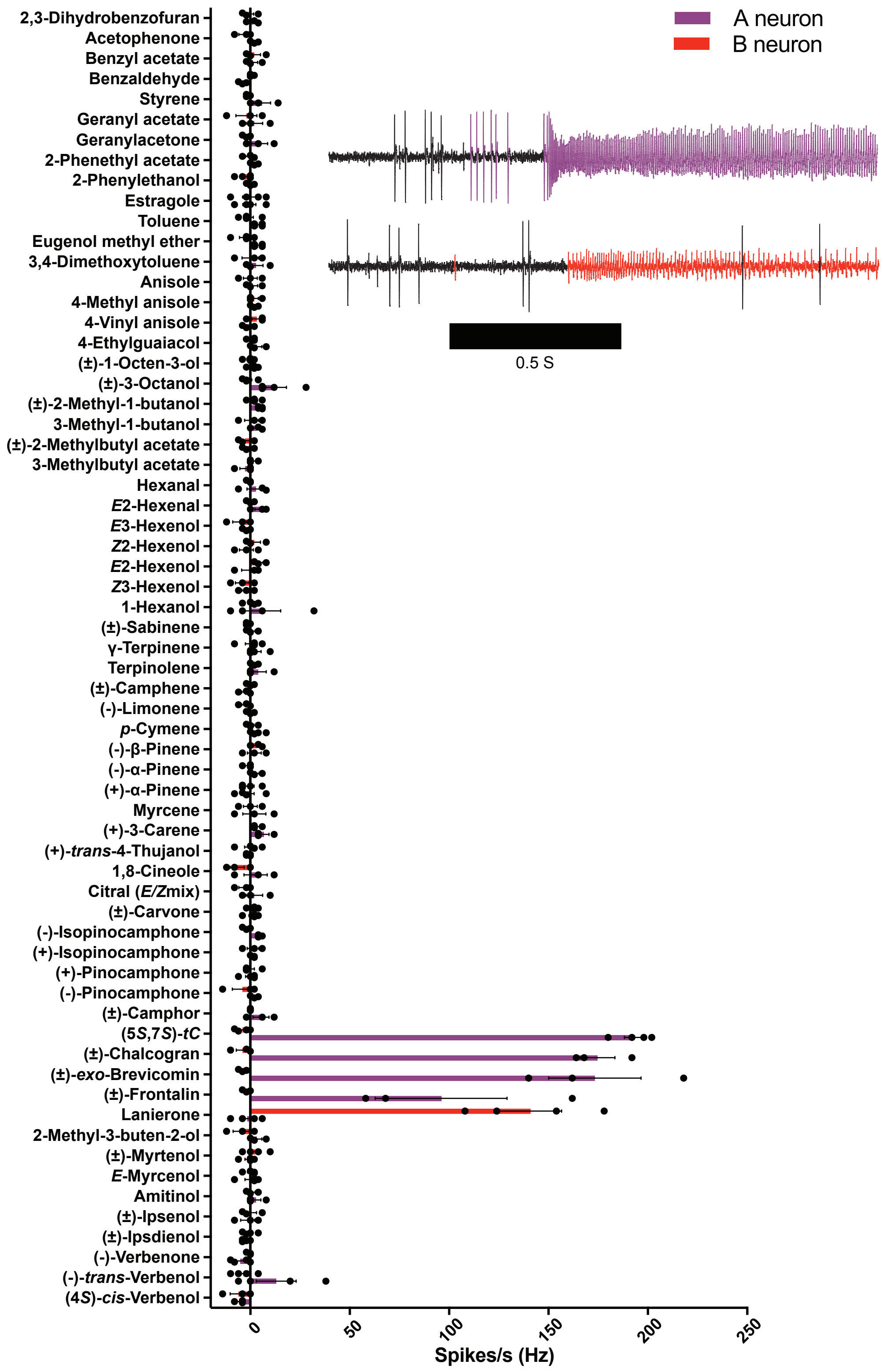

Supplementary Figure S6. Response spectra of the (5*S*,7*S*)-*trans*-conophthorin A-neuron OSN class (purple bars; N=3-4) and the co-localized lanierone-responsive B-neuron (red bars; N=3-4) at the 10 µg dose. Error bars show SEM; black dots represent individual data points. Representative action potential traces with response to (5*S*,7*S*)-*trans*-conophthorin in the A-neuron (large spike amplitude; purple spikes) and lanierone in the B-neuron (small spike amplitude; red spikes) are also shown. Raw data are reported in Additional file 2.

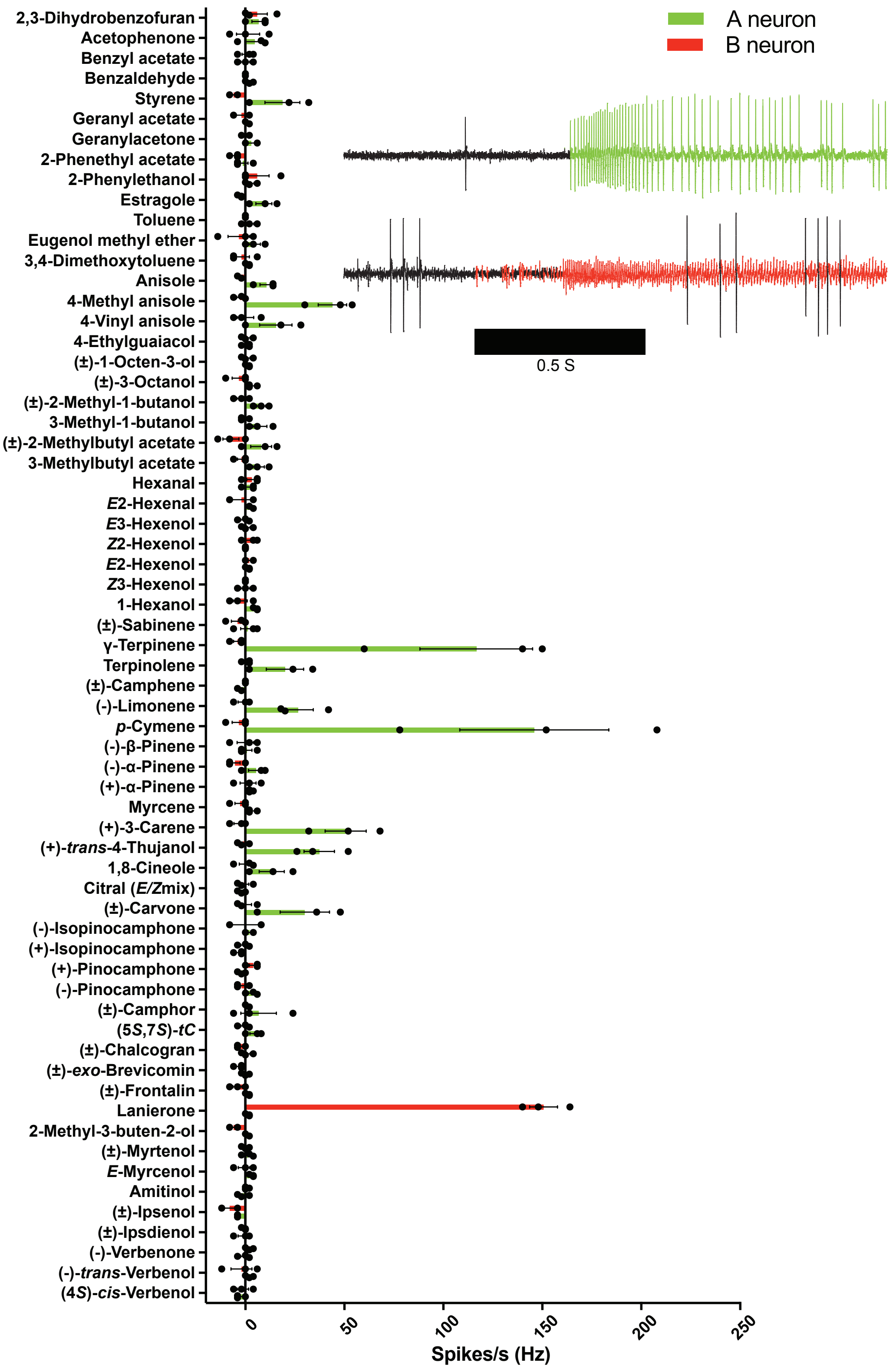

Supplementary Figure S7. Response spectra of the *p*-cymene A-neuron OSN class (green bars; N=3) and the co-localized lanierone-responsive B-neuron (red bars; N=3) at the 10 µg dose. Error bars show SEM; black dots represent individual data points. Representative action potential traces with response to *p*-cymene in the A-neuron (large spike amplitude; green spikes) and lanierone in the B-neuron (small spike amplitude; red spikes) are also shown. Raw data are reported in Additional file 2.

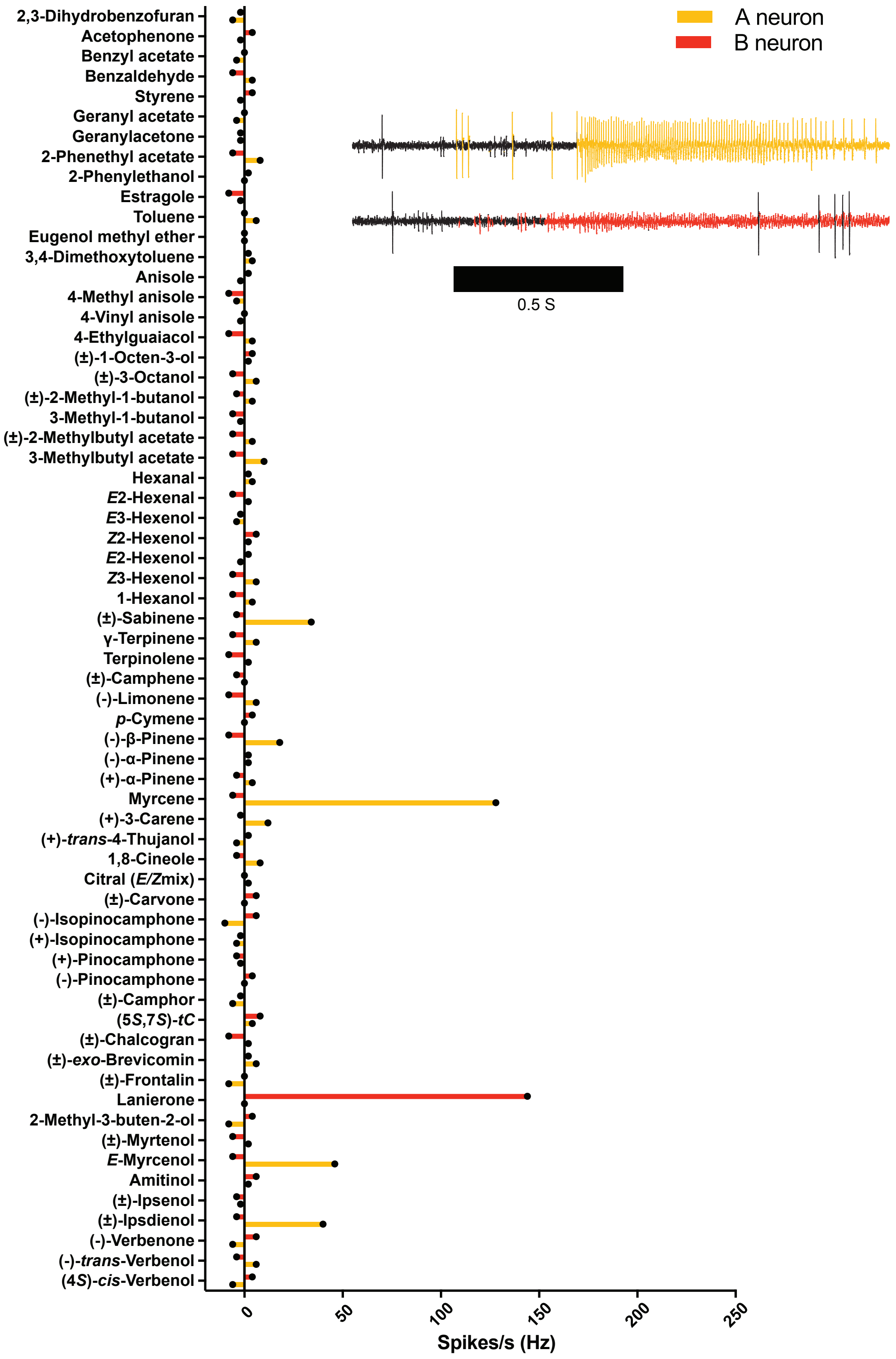

Supplementary Figure S8. Response spectra of the myrcene A-neuron OSN class (orange bars; N=1) and the co-localized lanierone-responsive B-neuron (red bars; N=1) at the 10 µg dose. Black dots represent individual data points. Representative action potential traces with response to myrcene in the A-neuron (large spike amplitude; orange spikes) and lanierone in the B-neuron (small spike amplitude; red spikes) are also shown. Raw data are reported in Additional file 2.
